# Minor temperature shifts do not affect chromosomal ploidy but cause transcriptomic changes in *Leishmania braziliensis* promastigotes *in vitro*

**DOI:** 10.1590/0074-02760190413

**Published:** 2020-04-27

**Authors:** Nathalia Ballesteros, Nubia M Vásquez, Luz H Patiño, Lissa Cruz-Saavedra, Juan David Ramírez

**Affiliations:** Universidad del Rosario, Facultad de Ciencias Naturales, Departamento de Biología, Grupo de Investigaciones Microbiológicas, Bogotá, Colombia

**Keywords:** Leishmania braziliensis, temperature shift, copy number variation, transcriptome profile, heat shock protein, amastin-like protein, ABC transporter

## Abstract

**BACKGROUND:**

The leishmaniases are complex neglected diseases caused by protozoan parasites of the genus *Leishmania*. *Leishmania braziliensis* is the main etiological agent of cutaneous leishmaniasis in the New World*.* In recent studies, genomic changes such as chromosome and gene copy number variations (CNVs), as well as transcriptomic changes have been highlighted as mechanisms used by *Leishmania* species to adapt to stress situations.

**OBJECTIVES:**

The aim of this study was to determine the effect of short-term minor temperature shifts in the genomic and transcriptomic responses of *L. braziliensis* promastigotes *in vitro*.

**METHODS:**

Growth curves, genome and transcriptome sequencing of *L. braziliensis* promastigotes were conducted from cultures exposed to three different temperatures (24ºC, 28ºC and 30ºC) compared with the control temperature (26ºC).

**FINDINGS:**

Our results showed a decrease in *L. braziliensis* proliferation at 30ºC, with around 3% of the genes showing CNVs at each temperature, and transcriptomic changes in genes encoding amastin surface-like proteins, heat shock proteins and transport proteins, which may indicate a direct response to temperature stress.

**MAIN CONCLUSIONS:**

This study provides evidence that *L. braziliensis* promastigotes exhibit a decrease in cell density, and noticeable changes in the transcriptomic profiles. However, there were not perceptible changes at chromosome CNVs and only ~3% of the genes changed their copies in each treatment.

Leishmaniases are caused by protozoan parasites of the *Leishmania* genus and involve a variety of clinical manifestations: cutaneous leishmaniasis (CL), mucocutaneous leishmaniasis and visceral leishmaniasis. These diseases are a major public health problem in 98 countries around the world, where 12 million people are infected, more than 350 million people are at risk of infection and 1.3 million new cases occur each year.^1^ CL is the most common clinical manifestation; between 0.7 and 1.3 million new cases of CL are reported annually and about 90% of them occur in Afghanistan, Algeria, Brazil, Iran, Pakistan, Peru, Saudi Arabia, Syria and Colombia. CL is also the most important and common clinical manifestation in the New World; it is characterised by ulcerative and deformative lesions, for which the most common causative species in the New World is *Leishmania braziliensis*.^1^ ^,^ ^2^

Temperature is a factor that determines whether the *Leishmania* parasite can develop appropriately. This was reported in a study by Hlavacova et al.^3^ in which they evaluated the effect of temperature on the life cycle of three *Leishmania* species inside two different species of vector (sandflies) and concluded that the response to temperature is species-specific. Other studies reported different results depending on the temperature shifts and the *Leishmania* species studied, some of the results showed an increase in parasite division, morphological changes (the parasites acquired a round shape), loss of motility,^4^ and changes in their differentiation rate or survival.^4^ However, there have been few studies aimed at understanding the biology of *Leishmania* when subjected to temperature stress. The majority of studies have focused on the impact of temperature shifts on the distribution of the vectors and the corresponding association with the number of leishmaniasis cases.^5^ Other studies have evaluated this variable in the several *Leishmania* species promastigotes in terms of the thermal shock associated with a change of host, in which the specific production of heat shock proteins (HSPs) occurs with an increase in temperature.^6^ ^,^ ^7^ ^,^ ^8^ However, little is known about the effect of temperature on the genomic and transcriptomic responses of *Leishmania*.

However, other studies on *Leishmania* have provided insights into the important role that genotypic plasticity plays in the response to different stress situations, with chromosome ploidy changes, amplifications and/or deletions of whole genes or chromosomes [copy number variations (CNVs)], being some of the mechanisms used by *Leishmania* to acquire resistance to antileishmanial drugs,^9^ ^,^ ^10^ to adapt to environmental conditions,^11^ or to distinguish different strains and species.^12^ Furthermore, some studies reported the impact of genomic changes on the transcriptome of *Leishmania*. For example, Dumetz et al.^13^ revealed the correlation between some chromosomal CNVs with the transcriptomic profile of the parasite, and Iantorno et al.^14^ showed that 85% of the differences in gene expression among *L. tropica* isolates could be explained by CNVs at the chromosomal and gene levels.

Despite awareness that *Leishmania* genotypic plasticity is an adaptation strategy to stress situations, the impact of temperature shifts on the genome and/or transcriptome of *Leishmania* parasites is poorly understood. In this study, we used next generation sequencing (DNA-seq and RNA-seq) to determine the possible genomic and transcriptome impacts that shifts in temperature may have on *L. braziliensis* promastigotes in the short-term *in vitro*. Our findings provide the first insights into the genomic and transcriptomic changes that might confer the short-term adaptive capacity of *Leishmania* to temperature stress, increasing awareness of the biology of this important parasite in the New World.

## MATERIALS AND METHODS

*Culture conditions and growth curves* - Promastigotes of *L. braziliensis* from strain MHOM/BR75/M2904 were cultivated in Roswell Park Memorial Institute (RPMI) (Sigma-Aldrich, MO, US) growth medium supplemented with 10% heat-inactivated foetal bovine serum (Invitrogen, CA, US). The parasites were incubated at four different temperatures, 24ºC, 26ºC, 28ºC and 30ºC, and the cultures at each of these temperatures were incubated with 5% CO_2_. A control temperature of 26ºC was included because it is a standard temperature for *in vitro* promastigote culture. We prepared cultures with three biological replicates per temperature with an initial concentration of 1 × 10^6^ parasites/mL. The parasite density for all culture replicates at each temperature was quantified using a Neubauer chamber for seven consecutive days. This quantification was used to construct growth curves for each temperature. From these curves, we determined the beginning of the logarithmic phase (BLP) by defining the day on which a significant difference emerged compared with the initial concentration. Statistical analyses were performed using the program GraphPad Prism (https://www.graphpad.com/scientific-software/prism/), where we analysed normality using the Kolmogorov-Smirnov test and subsequently used the Dunnett test of multiple comparisons to determine the day on which the logarithmic phase started. To determine whether there was a difference between the evaluated temperatures, we conducted a two-way ANOVA test comparing each treatment with the control temperature. In addition, we performed a Kruskal-Wallis test to determine whether the parasite density during the extraction day was significantly different between the treatments. P values < 0.05 were considered statistically significant.

**Fig. 1:**
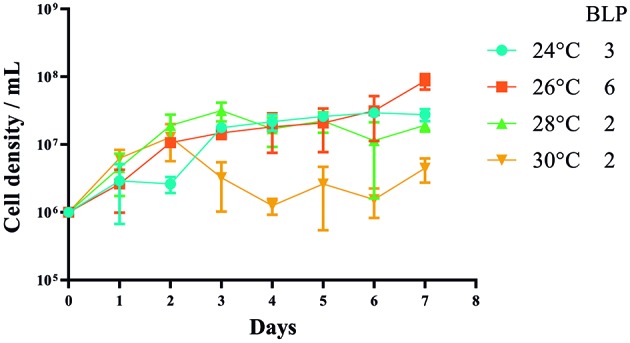
effect of temperature on the growth curve of *Leishmania braziliensis* promastigotes. Parasites were cultured at four different temperatures: 26ºC (orange), as the control temperature; 24ºC (light blue); 28ºC (light green); and 30ºC (yellow). The beginning of the logarithmic phase (BLP) for each treatment is indicated. Parasite growth was monitored daily for seven days through a Neubauer chamber. Bars represent standard errors obtained from three independent experiments. The cultures had an initial concentration of 1 × 10^6^ parasites/mL. The days of quantification are represented on the x-axis and the concentration of parasites per mL is expressed in a logarithmic scale on the y-axis.

*Isolation of RNA and DNA* - DNA and RNA extractions were performed on the day of the BLP, as defined by the parasite growth curves at each temperature. The DNA was extracted from one sample per treatment and was isolated using the Ultraclean Tissue and Cell DNA Isolation kit (MO BIO, CA, US), following the manufacturer’s standard protocol. Total RNA was extracted from two independent biological replicates, using the RNeasy Plus Mini Kit (Qiagen) following the manufacturer’s instructions. To control the sequencing process reliability, two technical replicates from each biological replicate were included. The concentration and quality of the DNA and RNA samples were quantified using a NanoDrop 2000 spectrophotometer (Thermo Fisher Scientific™), and the integrity was assessed by electrophoresis in a 1% agarose gel. All samples had A_260_/A_280_ ratios higher than 2.0. See Supplementary data **(Fig. 1)** for the integrity data and the A_260_/A_280_ values of all of the samples.

*Genome and transcriptome sequencing* - Once extracted, the DNA and RNA were sequenced by the Illumina HiSeq X-TEN system and libraries were prepared as follows: Microbial Mate-Paired for DNA and Strand-specific TrueSeq RNA-seq Library Prep (Illumina) for RNA with an insert size of 350 bp. The reads were 2 × 150 bp in length. The sequencing was performed by Novogene Bioinformatics Technology Co., Ltd., Beijing, China. The software FastQC (https://www.bioinformatics.babraham.ac.uk/projects/fastqc/) was used to determine the reliability of the sequencing.

*Mapping of DNA and RNA reads* - The reads were mapped to the reference genome *L. braziliensis* MHOM BR75 M2904 using the software SMALT v0.7.4 (www.sanger.ac.uk/resources/software/smalt/) with an exhaustive searching option -x and -y 0.8, a reference hash index of 13 bases and a sliding step of 3. We also mapped the reads with an identity threshold y = 0.8 to prevent mapping of non-*Leishmania* reads to the reference. Finally, read file merging, sorting and elimination of PCR duplicates were implemented with the software Samtools v0.1.18 (https://sourceforge.net/projects/samtools/) and Picard v1.85 (https://broadinstitute.github.io/picard/).

Genomic data analysis

*Evaluation of chromosome and gene copy number variations (CNVs)* - To obtain the read depth per chromosome, the sequencing data were normalised by the mean depth for the 35 chromosomes of *L. braziliensis*. The range of chromosome ploidy (P) was determined from the normalised read depths of the chromosomes and was defined as follows: p < 1.5 (haploid), 1.5 ≤ p < 2.5 (diploid), 2.5 ≤ p < 3.5 (triploid), 3.5 ≤ p < 4.5 (tetraploid) and 4.5 ≤ p < 5.5 (pentaploid). Also, we used vcf tools for calculating allelic chromosome frequency to evaluate and confirm ploidy results. The heatmaps were created using the R package Heatmap3. We compared the ploidy between temperatures and against control temperatures by calculating the p value using a Kruskal-Wallis test for independent samples.

To evaluate gene CNVs, the mean read per gene was determined, considering the possible impact of chromosome ploidy. The genes were filtered using the thresholds of a fold-change of Z score > 2 and p value < 0.05 compared with the control temperature of 26ºC. Then, the filtered genes were analysed according to the shared and unique genes identified at each temperature. For the supplementary figures of the read depth distribution of important chromosomes, we first calculated the depth with Samtools and then plotted the data with the program Gnuplot (http://www.gnuplot.info/). Data were classified into genomic locations and gene ontology (GO) terms were obtained through the free database Tritrypdb (http://tritrypdb.org/tritrypdb/), and the software Revigo was used to summarise the GO terms and remove any redundancy. We calculated the percentage of genes per ontology classification considering the total of genes with CNVs (i.e., with an increased or decreased copy number). We used a cut-off of > 5% of genes categorised to a specific GO term and all of the ontology terms had a p value of < 0.05. Statistical analysis was performed using the software GraphPad Prism. We performed tests of normality using the Kolmogorov-Smirnov and Shapiro-Wilks tests and then the Kruskal-Wallis test as a nonparametric test for independent samples to compare the read depths of each gene following different temperature shifts. For the GO analysis, we used terms incorporating a high percentage of genes; the percentages were calculated from the total of genes with CNVs (i.e., an increased or decreased copy number) compared with the control temperature. Finally, we constructed Venn diagrams, using Microsoft Office tools, taking into consideration the quantity of shared and unique genes with CNVs across the three temperatures.

*Single-nucleotide polymorphism (SNP) analysis* - To conduct single-nucleotide analysis, the SNPs, small insertions and deletions were called by the software Toolkit v3.4 GATK (https://software.broadinstitute.org/gatk/). Low-quality SNPs were filtered using GATK Variant Filtration, and Samtools was used to avoid false positives. The software SnpEff v4.1 (http://snpeff.sourceforge.net/) was used to classify the indels and SNPs according to their impact, such as being synonymous or nonsynonymous, or having a high or moderate impact in the genome.

*Transcriptome data analysis* - The levels of transcripts were quantified by assessing the read depth. The relative RNA-based ploidy (RNA-P) per chromosome was computed using the average read depth of transcripts and heatmaps were created using the R package Heatmap3. The ploidy range was the same as described previously in the DNA analysis section (2.5.1) and was based on the study by Rogers et al.^15^ The obtained results were statistically analysed through two-way snalysis of variance (ANOVA) (software GraphPad Prism) to determine whether each treatment was associated with a significant change in RNA compared with the results at 26ºC. To assess the impact of the gene CNVs in the transcriptomes, we calculated Spearman’s correlation coefficient using the software GraphPad Prism.

Differentially expressed genes (DEGs) were identified using DEseq 1.18.1 (R/Bioconductor); we used a fold-change cut-off of > 2 and a p value < 0.05 to define DEGs. The proportion of DEGs per chromosome was defined as follows: (number of DEGs per chromosome) / (number of total genes per chromosome) × 100. For the supplementary figure showing the read depth distribution of chromosomes with the highest number of up- or down-regulated genes, we first calculated the depth with Samtools and then plotted the data using the program Gnuplot (http://www.gnuplot.info/). Finally, GO information was extracted from a database (http://tritrypdb.org/tritrypdb/) using the option ‘biological process’ and a p value < 0.05 for the classification. We used Revigo software to summarise the GO terms and removed any redundancy. The Venn diagrams and GO figures were constructed using Microsoft Office tools, the GO analysis and its representation was performed through calculation of the percentage of genes associated with each GO term considering the total number of DEGs for each classification (up- and downregulated genes).

## RESULTS

*Growth curves of L. braziliensis promastigotes incubated in vitro under different temperatures* - At the control temperature, promastigotes reached the BLP on day 6, and no decrease in the cell density was observed in the control cultures (26ºC) over the course of the experiment (Fig. 1). Whereas at 24ºC, promastigotes reached the BLP on day 3, and similarly no decrease in the cell density was observed. After day 3, the number of parasites increased only slightly during the rest of the experiment (Fig. 1). The growth curves of the promastigotes at 28ºC and 30ºC were similar; in both cases, the BLP was on day 2, followed by a decrease in the cell density on the last days of the experiment (Fig. 1). Despite the similarity in pattern between these two highest temperatures, the magnitude of the cell density differed between them, with fewer parasites being observed at 30ºC (Fig. 1). Notably, the growth curves differed significantly at the different temperatures (p < 0.05). RNA and DNA extractions were performed on the BLP at each temperature, i.e., day 6 at 26ºC, day 3 at 24ºC, and day 2 at 28ºC and 30ºC.

**Fig. 2:**
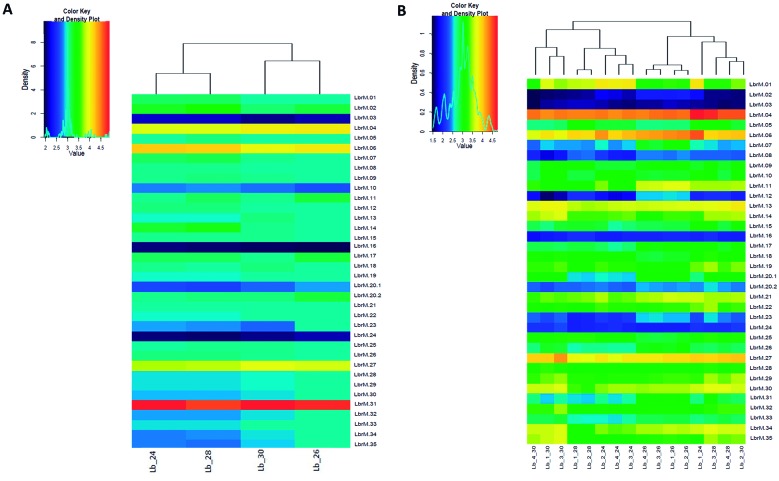
DNA-based and RNA-based ploidy values. (A) The DNA-based heatmap shows the ploidy of each of the 35 chromosomes calculated through the results obtained by high-throughput DNA sequencing. Each sample is indicated by the letters Lb (abbreviation for *Leishmania braziliensis*) and the temperature assessed. (B) The RNA-based heatmap shows the ploidy of each of the 35 chromosomes calculated through the results obtained by high-throughput RNA sequencing. Four replicates (two biological and two technical) are included and this is indicated between the letters Lb (abbreviation for *L. braziliensis*) and the last number (the temperature assessed); for example, Lb_1_30 is the first replicate at a temperature of 30ºC. The colour key indicates the chromosome ploidy value (p), which ranges from 1 to 5, and for which haploid is when p < 1.5, diploid 1.5 ≤ p < 2.5, triploid 2.5 ≤ p < 3.5, tetraploid 3.5 ≤ p < 4.5 and pentaploid 4.5 ≤ p < 5, as previously described. Both heatmaps include the three assessed temperatures along with the control temperature.

*Evaluation of copy number variations at the chromosomal level* - From the DNA sequencing data [the DNA-seq statistics are summarised in Supplementary data **(Table I)**], a comparison was made of the chromosomal ploidy of the samples at different temperatures (24ºC, 26ºC, 28ºC and 30ºC), which is illustrated in a heatmap in Fig. 2A. No significant difference in DNA ploidy was detected among the four temperatures evaluated (p = 0.975). This indicated that there was no change in ploidy of *L. braziliensis* promastigotes due to exposure to different temperatures over the short-term. In summary, three chromosomes (3, 16 and 24) were found to be disomic; chromosomes 4, 6 and 27 were tetrasomic; and chromosome 31 was pentasomic. The remaining 28 chromosomes were trisomic, as expected.^15^ To confirm the somy results, we calculated the allele frequency finding similar results as the ones obtained by mean normalisation depth. For example, the chromosome 7 is trisomic showing frequencies of 0,66 and 0,33; chromosome 27 is tetrasomic with allele frequency of 0,25 - 0,5 and 0,75, and chromosome 24 is disomic showing a frequency around 0,5; see Supplementary data **(Fig. 2)**.

Moreover, for each chromosome, we computed the mean transcript level to determine the ploidy value based on the results of RNA sequencing (RNA-P). Then, we compared the RNA-P with the DNA ploidy, as described above. We did not observe a difference in ploidy between the control and the treatments in any of the chromosomes (Fig. 2A). We also observed no difference in ploidy based on RNA-P (Fig. 2B). When comparing the control with each treatment using two-way ANOVA, the following p values were obtained: 0.073 (control (26ºC) vs. 24ºC), 0.220 (control vs. 28ºC) and 0.144 (control vs. 30ºC).

*Evaluation of the copy number variations (CNVs) at the gene level* - Gene CNVs were evaluated by comparing the results obtained at each tested temperature (24ºC, 28ºC and 30ºC) with those obtained at the control temperature (26ºC). From a total of 8507 genes, 253 genes (2.97%) at 24ºC, 247 genes (2.9%) at 28ºC and 260 genes (3.05%) at 30ºC presented CNVs (-2 > Z score > 2, equivalent to p < 0.05). While 61.33% ± 1.5% of the genes with CNVs at each temperature showed an increase, the remaining 38.67% ± 1.5% showed a decrease compared with the control. Furthermore, mean values of 41.84% ± 1.7% of the genes with an increased copy number and 32.6% ± 3.6% of the genes with a decreased copy number encoded hypothetical proteins at each temperature assessed.

**Fig. 3:**
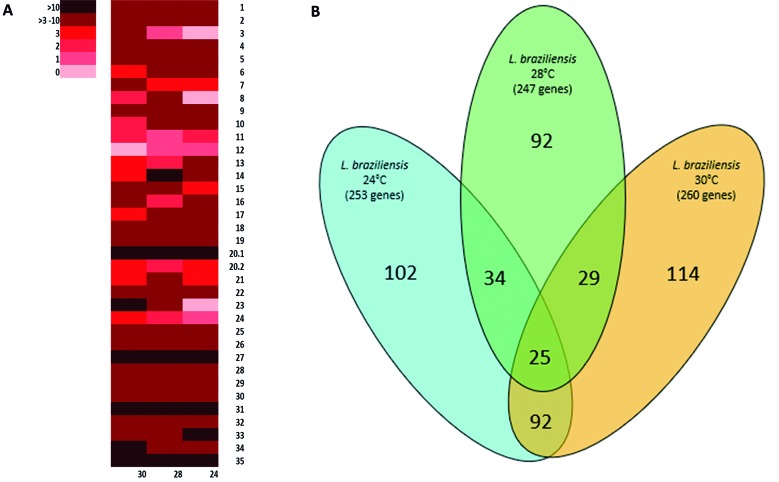
(A) Heatmap representing the number of genes per chromosome with copy number variations (CNVs) compared with the control. The coloured box indicates the ranges of genes with CNVs. The number below the heatmap indicates the temperature tested and the numbers on the right indicate each chromosome. (B) Venn diagram shows the number of genes with CNVs compared with the control and the number of overlapping genes of *Leishmania braziliensis* under different temperatures: 24ºC (light blue), 28ºC (light green) and 30ºC (yellow), with a fold change of Z score > 2 compared with the copy number at the control temperature (26ºC). The numbers in brackets are the total number of genes with CNVs at each temperature.

**Fig. 4:**
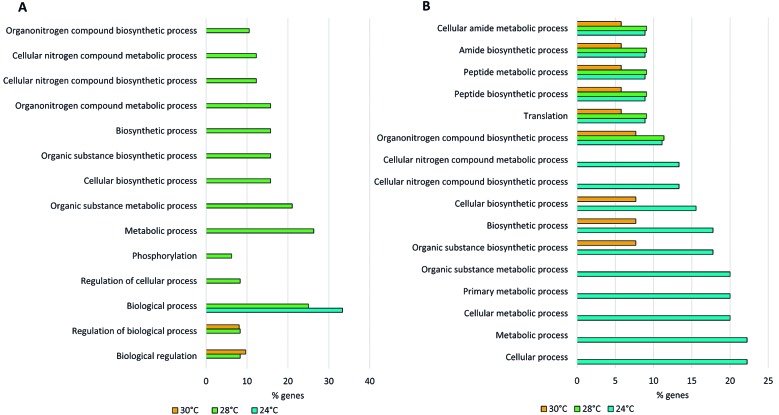
percentage of genes per ontology term for each temperature based on the DNA sequencing results. Unique genes with a fold-change in the copy number of 2 compared with the control (at 26ºC) (control temperature) were evaluated for each temperature: 24ºC (102 genes), 28ºC (92 genes) and 30ºC (114 genes), to obtain the ontology terms. Only the first 10 terms were used for each temperature. We calculated the percentage considering the total number of genes with CNVs for each case and determined the decreased and increased copy numbers. (A) The ontology of the increased read depth compared with the control. (B) The ontology of the decreased read depth compared with the control. The x-axis corresponds to the percentage of genes classified for an ontology term (the percentage was calculated from the total number of genes in the category of temperature and the increased or decreased read depth).

**Fig. 5:**
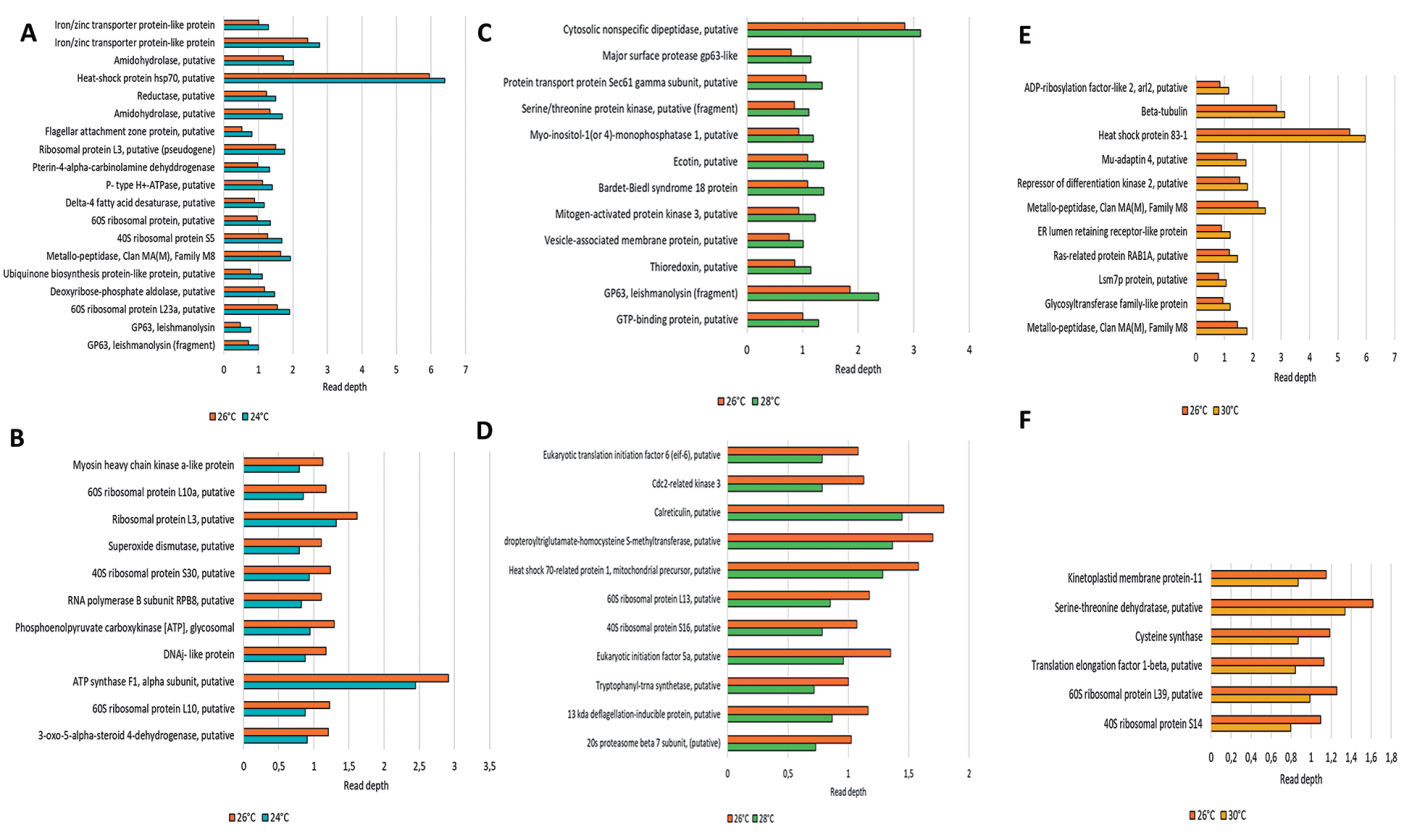
read depth of genes compared with those at the control temperature based on the DNA sequencing results. The ontology classifications of these genes are illustrated in Fig. 4. The genes with increased read depth compared with the control at each temperature are presented in the upper three graphs in the figure (A, C, E), while the genes with a decreased read count are shown in the lower three graphs in the figure (B, D, F). Joined genes are shown in the Supplementary data (Figs 2, 3).

**Fig. 6:**
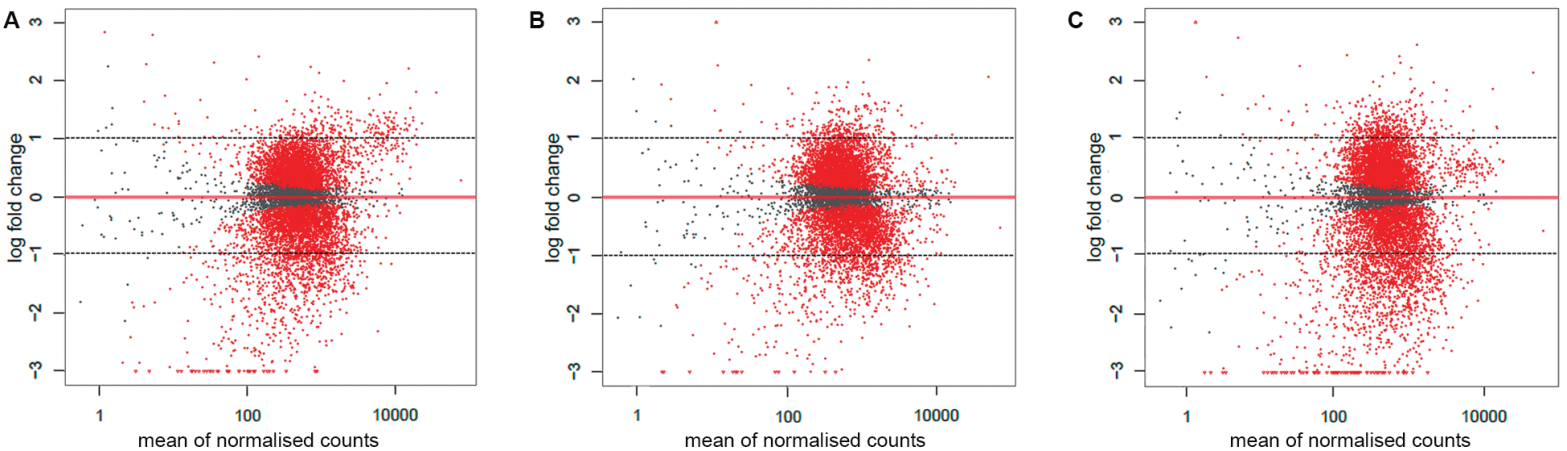
MA plots representing the differentially expressed genes (DEGs) of *Leishmania braziliensis* promastigotes under each treatment, (A) at 24ºC, (B), 28ºC and (C) 30ºC. The up- and downregulated DEGs are highlighted in red and the genes with steady-state levels of RNA are represented by grey dots. The dotted black lines represent the cut-off of the fold change (log fold change > 1 and < ˗1).

Supplementary data **(Fig. 3)** shows the distribution of genes with a fold-change of 2 in CNVs for each temperature compared with the level for the control temperature (26ºC). For all temperatures, the chromosomes with more than 10 genes with CNVs were chromosomes 14, 20.1, 27, 31 and 35 (Fig. 3A). The read depth distributions per chromosome showing the changes per position are presented in Supplementary data **(Figs 4, 5, 6)**, and chromosome 31 possessed the highest number of genes with CNVs across the three temperatures [Supplementary data **(Tables II-IV)**]. In terms of the percentage of genes showing CNVs per chromosome, the highest rate was detected for chromosome 2. In this chromosome, all of the genes with CNVs at 24ºC showed an increase in their copy number compared with the control temperature.

**Fig. 7:**
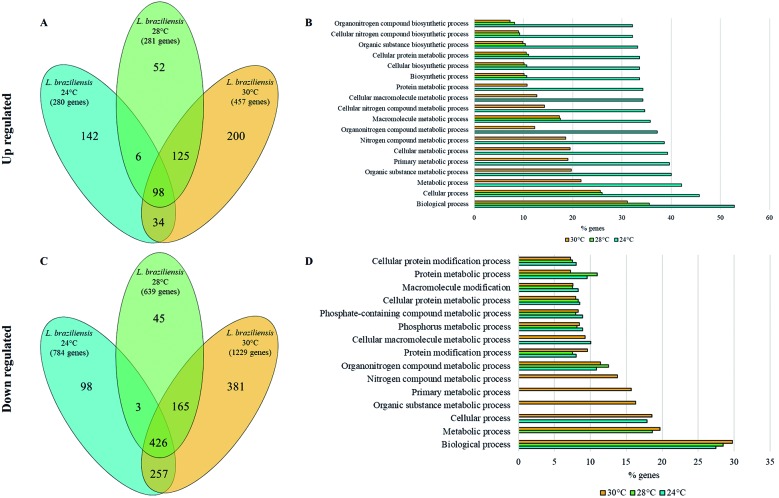
venn diagrams and gene ontology terms of the differentially expressed genes (DEGs). Illustrations of the number of DEGs shared between two or three temperature treatments and the number of unique DEGs at each temperature assessed for both DEG classifications: (A) upregulated and (C) downregulated. The numbers in brackets are the total number of DEGs at each temperature. Representation of the gene ontology (GO) terms with the highest proportion of genes associated with them; GO terms for (B) upregulated and (D) downregulated genes. The x axis shows the percentage of genes associated with each GO term calculated considering the total number of DEGs for each classification (up- and downregulated genes). Each colour represents a temperature, 24°C (light blue), 28°C (light green) and 30°C (yellow).

Among the three tested temperatures, 92 genes shared CNVs compared with the control (Fig. 3B), and the variations in these genes (i.e., increased or decreased copy number) were consistent across the temperatures. There was also no significant difference in the read depth of the shared genes (p = 0.971 for the genes with increased read depth and p = 0.982 for the genes with decreased read depth). Among the shared genes [Supplementary data **(Fig. 7)**] that increased in copy number compared with the control were genes that encode elongation factor 1-alpha (LbrM.17.0090) and a surface antigen protein (LbrM.04.1330). Those with decreased copy number included genes that encode an amastin-like surface protein and a NADH-dependent fumarate reductase (LbrM.34.1110). Moreover, some genes that encode amastin-like proteins (LbrM.08.1060, LbrM.18.0460) and beta and alpha tubulins increased their copy number, while others that encode similar proteins decreased their copy number (LbrM.13.0200, LbrM.33.0920, LbrM.33.0950). The genes for which variations were only shared between two temperatures are presented in Supplementary data **(Fig. 8)**. At 28ºC and 30ºC, more genes showed a decrease in copy number and these included genes that encode a ubiquitin-conjugating enzyme E2 (LbrM.02.0420), tRNAs (LbrM.20.1.tRNA8, LbrM.23.tRNA10, LbrM.23.tRNA6, LbrM.23.tRNA8) and an amastin surface protein (LbrM.24.1590). At 24ºC and 28ºC, more genes showed an increase in copy number, such as those encoding a peptidase M20/M25/M40 (LbrM.33.2100) and a receptor-type adenylate cyclase (LbrM.17.0110). Finally, between 24ºC and 30ºC, fewer genes were shared, but the genes that encode HSP83-1 and HSP70 (LbrM.33.0330, LbrM.28.2970) showed a decreased copy number. The number of unique genes totalled 114 genes for 30ºC, 102 genes for 24ºC and 92 genes for 28ºC (Fig. 3B). For these genes, we obtained the associated ontology terms through the database tritrypdb.org, choosing the option ‘biological process’ to determine the function of the genes.

Fig. 4 shows the ontology terms associated with large numbers of genes, all of the GO terms had a p value < 0.05. Some ontology terms were shared between unique genes with an increased copy number at each temperature. For example, genes with CNVs at 24ºC and 28ºC shared the ontology term ‘biological process’ (Fig. 4A), whereas those with CNVs at 30ºC and 28ºC shared ontology terms such as ‘biological regulation’ and ‘regulation of biological process’ (Fig. 4A). Numerous unique ontology terms were associated with genes with CNVs at 28ºC including ‘phosphorylation’, ‘regulation of cellular process’, ‘organonitrogen compound biosynthetic process’ and ‘cellular nitrogen compound metabolic process’ (Fig. 4A). Otherwise, the ontology terms for genes that showed a decreased copy number differed from those associated with genes showing an increased copy number. Some ontology terms that were shared among the three treatments for genes with decreased copy numbers were ‘amide and peptide metabolic and biosynthetic process’ and ‘organonitrogen compound biosynthetic process’ (Fig. 4B). The ontology terms only shared by genes following treatment at 30ºC and 24ºC were ‘organic substance metabolic process’, ‘cellular biosynthetic process’ and ‘biosynthetic processes’, and the unique terms at 24ºC were related to metabolic, cellular and biosynthetic processes (Fig. 4B).

Next, we investigated the genes associated with the ontologies described above. Fig. 5 shows the genes with an increase in copy number compared with that at the control temperature. At 24ºC, the gene with the highest read depth encoded HSP70, putative protein (LbrM.28.2990), followed by an iron/zinc transporter-like protein (LbrM.31.3480). At 28ºC, one gene with an increased read depth was that encoding GP63 leishmanolysin (LbrM.10.1710) and one gene with a decreased read depth was heat shock 70-related protein 1 (LbrM.30.2420). At 30ºC, the notable genes showing an increased copy number were heat shock protein 83-1 (LbrM.33.0340) and beta-tubulin (LbrM.33.0930). By contrast, genes showing a decreased copy number encoded a serine-threonine dehydratase (LbrM.06.0720) and translation elongation factor 1-beta (LbrM.35.1570).

Finally, we evaluated a total of 38,703 SNPs and 44 (0.11%) had a high impact and 7611 (17.33%) had a moderate impact on DNA. Among the three temperatures evaluated, there were no significant differences in the SNPs (p = 0.947). We also evaluated a total of 18,755 indels, and these indels did not differ significantly among the three temperatures (p = 0.939).

*Evaluation of copy number variations (CNVs) at the gene level and the impact on the transcriptome profiles* - After assessing the changes in gene copy number, we evaluated their impact at the transcriptome level. First, to establish whether there was a correlation between the increase/decrease in copy number and the increase/decrease in gene expression, we calculated Spearman’s correlation coefficient. The results indicated no correlation between CNVs and gene expression in all of the treatments, since the *r* values were near zero and the *p* values were > 0.05. One example of this lack of correlation was on chromosome 14 at 24ºC and 28ºC, in which all of the genes with CNVs showed an increase in their copy number, but some genes such as LbrM.14.1110 and LbrM.14.1310 showed a decrease in RNA synthesis compared with the control. Despite this, we examined the genes with an increase in both their copy number and expression, as well as those with a decrease in their copy number and expression, revealing 15 and 13 genes at 24ºC, 9 and 14 genes at 28ºC, and 11 and 21 genes at 30ºC, respectively.

Interestingly, a gene encoding an elongation factor (LbrM.17.0090) showed an increase in copy number and expression under the three temperatures. Furthermore, we found other genes that encode a hypothetical protein (LbrM.04.0230) and an alpha-tubulin (LbrM.29.2700) that increased their copy number and expression at 24ºC and 28ºC. By contrast, some genes decreased their copy number and expression under the three treatments: two encoding amastin-like surface proteins (LbrM.18.0460 and LbrM.180470), which are also tandem genes, and one encoding an NADH-dependent fumarate reductase gene (LbrM.34.1110). At 24ºC and 30ºC, another gene that encodes an NADH-dependent fumarate reductase (LbrM.34.1100) exhibited a decreased copy number and expression; at 28ºC and 30ºC, genes that encode amastin-like surface protein (LbrM.24.1590) and a beta-tubulin (LbrM.33.0990) also showed these decreases; whereas at 24ºC and 28ºC, only one gene that encodes a poly-zinc finger protein (LbrM.35.1790) shared this behaviour. Here, it is worth noting that, at 30ºC, there were two genes of particular interest that could have changed their expression as a direct response to the temperature. One of these genes encodes a stress-inducible protein (LbrM.35.0120), which increased its copy number and expression; while the other encodes a multidrug resistance protein (LbrM.35.1520), which showed decreases in both its copy number and expression.

*Differentially expressed genes (DEGs) in promastigotes at each assessed temperature* - We evaluated the changes in the transcription profiles of four replicates, two biological and two technical, in response to the different temperatures at which *L. braziliensis* promastigotes were incubated. The total number of reads obtained for the RNA sequences, as the number of aligned reads per treatment, are represented in Supplementary data **(Table V)**. We compared the gene expression levels with the results obtained at the control temperature (26ºC), obtaining log-fold changes. The cut-off was chosen as a fold change (FC) > 2 (log-fold change > 1), as indicated by the dotted line in Fig. 6. At the three temperatures, similar patterns of DEGs were identified, with all of them including more downregulated genes than upregulated genes (Fig. 6). The promastigotes incubated at 28ºC had 920 genes with FC > 2 and p < 0.05 (11.2%) from a total of 8204 DEGs; of these 920 genes, 639 were downregulated and 281 were upregulated. By contrast, the promastigotes incubated at 24ºC had 1064 genes with FC > 2 and p < 0.05 (13%), from a total of 8205 DEGs; of these 1064 genes, 784 were downregulated and 280 were upregulated. Finally, in order of lowest to highest number of DEGs, at 30ºC the promastigotes showed changes in the expression of 8207 genes, of which 1686 had FC > 2 and p < 0.05 (20.5%); these were divided into 1229 downregulated and 457 upregulated genes (see the Supplementary Dataset for the complete list of transcripts with their fold-changes and p values).

We also calculated the percentage of DEGs per chromosome for the upregulated and downregulated genes in the three treatments. Interestingly, we found that chromosome 12 had the highest percentage of downregulated genes at temperatures of 24ºC and 30ºC (the treatments with the highest numbers of DEGs), with values of 21.3% and 28.7%, respectively. In other words, chromosome 12 was the most affected at these temperatures, as shown by the downregulation of a large proportion of its genes. Whereas, the chromosome with the highest proportion of upregulated genes at 30ºC (the treatment with the most DEGs) was chromosome 25, with 9.3% of genes upregulated [Supplementary data **(Fig. 9)**].

After categorising the DEGs into two categories, up- and downregulated genes, we performed an analysis to determine the ontology of these genes. In this way, we found that the three treatments had similar patterns since a large number of genes encode hypothetical proteins, followed by fewer domains, families and proteins of unknown function. Another observed pattern was that the downregulated genes were associated with fewer GO terms than the upregulated genes, even though the number of downregulated genes was greater. Thus, we concluded that the large number of downregulated genes had similar or redundant biological functions, mostly associated with biological processes, organonitrogen compound metabolic processes and protein metabolic processes, among others (Fig. 7D).

In Fig. 7B and 7D, we illustrate the GO terms with the maximum proportion of up- and downregulated genes associated with them for the three assessed temperatures. All of the GO analyses generated a p value < 0.05. In the case of the upregulated genes, we observed a pattern in which, at 24ºC, a great proportion of genes were associated with all of the GO terms represented in Fig. 7B, so, the upregulation of a superior number of genes associated with biological, cellular, biosynthetic and metabolic processes at 24ºC compared with the number of genes associated with these same GO terms at other temperatures may be the reason why parasites under 24ºC were less affected in terms of growth and cellular concentration (Fig. 1).

Despite the large number of DEGs associated with metabolic, biological and cellular processes, a small proportion of other genes were associated with GO terms related to responses to stimuli; for example, some genes that were upregulated at 28ºC and 30ºC were related to responses to external stimuli. Furthermore, some genes related to transmembrane transport at 30ºC were up- or downregulated, with downregulation being predominant.

*The differentially expressed genes were unique at each temperature* - As we described above, numerous GO terms were shared among the different temperature treatments; this is in part a consequence of the DEGs shared between treatments (Fig. 7A,C). Next, we evaluated the individual DEGs that were unique for each temperature and the DEGs shared between two or three temperatures. We concluded that the DEGs uniquely up- or downregulated following each treatment were expressed as a direct response to the temperature change.

We evaluated the unique DEGs for both up- and downregulated genes in the three treatments, and the 10% of genes with the largest change in their expression were compared with the expression at 26ºC (log-fold change). At 24ºC, the gene with the largest log-fold change (2.834) encoded a protein kinase (LbrM.34.1040), followed by other genes encoding ribosomal genes (Table I) and a gene encoding an activator of HSP90 ATPase (LbrM.18.0230), which showed an increase in expression with a log FC of 1.138 and a p value = 3.261E˗103 (data not shown, see Supplementary Dataset). By contrast, among the genes downregulated at this temperature, the gene with the largest decrease in expression was encoded by an amastin-like surface protein (with a log FC of ˗2.815), followed by a gene cluster that encodes a tuzin-like protein (LbrM.20.2420) and another amastin-like surface protein (LbrM.20.2410) (Table I).

**TABLE I:** Up- and downregulated differentially expressed genes (DEGs) expressed only at 24ºC. The selection was based on those genes with the highest 10% of log fold change in their expression compared with the control. The cut-offs were fold change > 2 (log fold change > 1) and p value < 0.05

Gene ID	Product description	Log2 fold change	p value
LbrM.34.1040	Protein kinase, putative	2.834	0.048
LbrM.02.0010	Phosphoglycan beta 1.3 galactosyltransferase 3	1.643	0.027
LbrM.05.0800	Methylthioadenosine phosphorylase, putative	1.541	2.907E-34
LbrM.35.3090	40S ribosomal protein S24e	1.531	4.915E-78
LbrM.21.1300	40S ribosomal protein S23, putative	1.504	1.071E-09
LbrM.28.1080	Ribosomal protein S20, putative	1.502	6.245E-76
LbrM.28.1100	Ribosomal protein S20, putative	1.474	7.291E-14
LbrM.10.1070	Histone H3	1.465	4.3318E-58
LbrM.11.0760	40S ribosomal protein S5	1.444	3.744E-29
LbrM.35.3980	60S ribosomal protein L34, putative	1.442	8.771E-33
LbrM.31.tRNA1	tRNA-Ala	1.431	0.002
LbrM.32.2950	Ribosomal protein L27, putative	1.426	2.335E-05
LbrM.06.0590	60S ribosomal protein L23a, putative	1.411	4.142E-23
LbrM.34.3760	60S ribosomal protein L27A/L29, putative	1.386	6.055E-71
LbrM.20.4310	Amastin-like surface protein, putative	-2.815	1.406E-107
LbrM.20.2420	Tuzin-like protein	-2.056	7.607E-20
LbrM.20.2410	Amastin-like surface protein, putative	-1.639	2.823E-45
LbrM.20.4290	Amastin-like surface protein, putative	-1.616	1.437E-47
LbrM.18.0060	Hypothetical protein, conserved	-1.567	1.415E-50
LbrM.28.2110	Zinc transporter 3, putative	-1.535	5.914E-11
LbrM.32.0330	Serine/threonine-protein kinase Nek1-related, putative	-1.482	3.978E-31
LbrM.12.0160	Cell division protein kinase, putative	-1.475	2.165E-36
LbrM.18.1000	Nrap protein, putative	-1.468	0.001
LbrM.32.3711	Hypothetical protein, conserved	-1.466	2.869E-23

By contrast, the genes that were upregulated only at 28ºC were found to be associated principally with transcriptional and translational processes, as indicated in Table II. Among the 10% most upregulated genes, the majority were involved in these processes. Whereas among the downregulated genes, across all of the treatments, we also observed the downregulation of a gene that encodes an amastin-like surface protein (LbrM.24.1600) (Table II) and the downregulation of other genes principally associated with metabolic processes.

**TABLE II:** The 10% of genes with the highest log fold change in their expression compared with the control. Among the up- and downregulated differentially expressed genes (DEGs) only expressed at 28ºC. The cut-offs were fold change > 2 (log fold change > 1) and p value < 0.05

Gene ID	Product description	Log2 fold change	p value
LbrM.11.tRNA5	tRNA-Arg	1.485	0.000
LbrM.34.4080	Splicing factor 3B subunit 10 (SF3b10), putative	1.428	7.672E-36
LbrM.13.1240	Ran-binding protein 1, putative	1.338	2.527E-13
LbrM.32.0060	Nuclear segregation protein, putative	1.261	4.836E-12
LbrM.01.0350	Hypothetical protein, conserved	1.245	3.458E-08
LbrM.16.0010	Anti-silencing protein a-like protein	1.224	5.478E-16
LbrM.04.0420	Hypothetical protein	-3.243	0.007
LbrM.23.snRNA2	Small nuclear RNA, U3 snRNA	-3.052	0.002
LbrM.24.1600	Amastin-like surface protein-like protein	-2.369	0.002
LbrM.31.3640	Phosphoglycan beta 1.3 galactosyltransferase 5 (fragment)	-1.894	0.038
LbrM.08.0700	SLACS	-1.690	2.220E-05

Similarly, of the 200 genes uniquely overexpressed at 30ºC (Fig. 7A), the majority were related to metabolic, biosynthetic, transcriptional and translational processes, as shown in Table III. In this table, the genes with the highest log FC values encoded tRNA-Gly and tRNA-Asn. Although the promastigotes at 30ºC exhibited upregulation of four genes directly associated with the stress response generated by the increased temperature, these included two encoding HSPs [heat shock 70-related protein (LbrM.30.2430, log FC = 1.154 and p = 2.40E-24) and HSP DNAJ (LbrM.27.0500, log FC = 1.059 and p = 1.485E˗21)] and two encoding DNAJ domain-containing proteins (LbrM.17.0050, log FC = 1.169 and p = 1.049E-20; and LbrM.32.0670, log FC = 1.054 and p = 1.124E-22), data not shown, see Supplementary Dataset. By contrast, among the downregulated genes at 30ºC, a gene encoding an ABC transporter (LbrM.27.1050, log FC = ‒1.35 and p = 5.99E-07) was detected.

**TABLE III:** The up- and downregulated differentially expressed genes (DEGs) with the 10% highest values in their expression compared with the control expressed only at 30ºC. The cut-offs were fold change > 2 (log fold change > 1) and p value < 0.05

Gene ID	Product description	Log2 fold change	p value
LbrM.11.tRNA3	tRNA-Gly	4.669	0.003
LbrM.10.tRNA2	tRNA-Asn	2.062	0.034
LbrM.27.0490	Hypothetical protein, conserved	1.587	4.756E-13
LbrM.16.1460	Trafficking protein particle complex subunit-like protein	1.416	6.804E-30
LbrM.34.3560	Methyltransferase domain-containing protein, putative	1.396	5.593E-83
LbrM.20.0220	CS domain-containing protein, putative	1.393	8.896E-55
LbrM.20.1400	Hypothetical protein, conserved	1.393	1.831E-27
LbrM.02.0140	Hypothetical protein, conserved	1.390	2.781E-35
LbrM.34.1830	Hypothetical protein, unknown function	1.386	2.703E-54
LbrM.20.3420	Ribosomal protein L14, putative	1.357	2.475E-20
LbrM.09.1410	Hypothetical protein, conserved	1.353	2.370E-28
LbrM.06.0130	Hypothetical protein, conserved	1.353	5.338E-25
LbrM.30.2970	Hypothetical protein, conserved	1.309	1.168E-19
LbrM.20.0820	Serine/threonine-protein phosphatase PP1, putative	1.307	6.336E-68
LbrM.29.1820	Histone H2A, putative	1.301	2.6739E-30
LbrM.04.1160	Hypothetical protein, conserved	1.297	9.194E-36
LbrM.28.0950	Hypothetical protein, conserved	1.296	8.645E-34
LbrM.27.0170	SET domain-containing protein, putative	1.291	1.269E-34
LbrM.12.0690	Hypothetical protein, conserved	1.287	3.706E-28
LbrM.34.4350	Zinc-binding domain-containing protein, putative	1.282	2.449E-19
LbrM.33.2020	Macrophage migration inhibitory factor-like protein	-2.464	1.617E-07
LbrM.05.1210	Surface antigen-like protein	-2.300	2.750E-12
LbrM.34.0520	Proteophosphoglycan ppg3, putative (fragment)	-2.218	2.398E-19
LbrM.10.0380	Folate/biopterin transporter, putative	-2.146	2.157E-13
LbrM.01.0720	Protein kinase, putative	-2.142	4.29E-22
LbrM.31.3030	Hypothetical protein, conserved	-2.084	1.483E-17
LbrM.14.0540	Hypothetical protein, unknown function	-1.987	5.258E-07
LbrM.13.0100	SURF1 family, putative	-1.987	1.669E-13
LbrM.01.0260	Long-chain-fatty-acid-CoA ligase, putative (fragment)	-1.937	2.211E-13
LbrM.30.0340	Hypothetical protein, conserved	-1.919	5.127E-15
LbrM.34.4200	Hypothetical protein, unknown function	-1.906	2.559E-24
LbrM.31.1450	Pyrophosphate-energized vacuolar membrane proton pump 1, putative	-1.870	5.926E-16
LbrM.29.2710	Hypothetical protein, conserved	-1.797	3.238E-17
LbrM.20.5790	TBC1 domain family member 20/GTPase, putative	-1.718	3.011E-08
LbrM.22.0010	CLN3 protein, putative	-1.716	1.25E-16
LbrM.27.1000	Protein of unknown function (DUF1295), putative	-1.669	2.388E-31
LbrM.23.1890	COG4 transport protein, putative	-1.660	3.379E-17
LbrM.18.0650	RNA binding protein, putative	-1.642	1.583E-16
LbrM.20.5530	Small myristoylated protein 1	-1.630	1.489E-13
LbrM.34.2530	Hypothetical protein, unknown function	-1.616	5.257E-14
LbrM.04.0790	Hypothetical protein, conserved (fragment)	-1.602	3.788E-14
LbrM.20.5760	Hypothetical protein, conserved	-1.595	2.345E-18
LbrM.35.3160	Phosphatidylinositol 3- and 4-kinase, putative	-1.587	2.433E-65
LbrM.24.0470	Protein of unknown function (DUF3184), putative (fragment)	-1.584	6.276E-37
LbrM.33.2090	Dual-specificity protein kinase, putative	-1.582	6.528E-16
LbrM.05.1110	DNA-directed RNA polymerase I largest subunit	-1.576	2.091E-07
LbrM.08.0450	Hypothetical protein, conserved	-1.570	5.701E-61
LbrM.05.0640	Hypothetical protein, conserved	-1.568	2.325E-11
LbrM.34.1560	Hypothetical protein, conserved	-1.549	2.463E-20
LbrM.03.0030	Hypothetical protein	-1.547	7.055E-17
LbrM.31.0510	Calpain-like protein 2	-1.543	1.024E-22
LbrM.34.2640	Galactokinase-like protein	-1.541	2.970E-31
LbrM.16.1480	Paraflagellar rod protein 2C	-1.538	4.496E-20
LbrM.03.0300	Hypothetical protein	-1.535	1.260E-39
LbrM.05.1130	Hypothetical protein, conserved	-1.531	2.306E-07
LbrM.05.1170	Hypothetical protein, conserved	-1.528	1.149E-22
LbrM.03.0770	Hypothetical protein, conserved	-1.526	4.082E-25
LbrM.12.0350	Myotubularin-related protein, putative	-1.523	2.390E-40

*Differentially expressed genes shared between temperatures* - As well as evaluating the genes expressed uniquely at each temperature, we also evaluated which genes were changed in expression in response to two or all of the test temperatures to determine which genes were up- or downregulated irrespective of the extent of the temperature shift. Among the genes that were altered in expression at all three temperatures, we found downregulated genes that encoded the same products as previously reported genes, such as those encoding (1) amastin-like surface proteins (LbrM.18.0460, LbrM.18.0470, LbrM.13.1330, LbrM.10.1520, LbrM.08.0670, LbrM.08.0680, LbrM.20.0950, LbrM.20.0960, LbrM.20.1080, LbrM.20.4340, LbrM.24.1590, LbrM.35.4370 and LbrM.35.4380), which in this case were overrepresented compared with previous results; (2) ABC transporters (LbrM.02.0350, LbrM.11.1040, LbrM.11.1020 and LbrM.11.0960); and (3) DNAJ domain protein (LbrM.24.1630), also known as HSP40.^8^

Among the DEGs shared between the 24ºC and 30ºC treatments, one gene was upregulated that encodes a stress-inducible protein, STI1 (LbrM.35.0120), with a similar log FC at both temperatures (1.044 and 1.069, respectively, see the Supplementary Dataset). Among the downregulated genes at these same temperatures, genes encoding ABC transporters (LbrM.06.0010, LbrM.11.1000, LbrM.11.1010 and LbrM.15.0930), an amastin surface glycoprotein (LbrM.28.1210) and heat shock 70-related protein 1 (LbrM.30.2450) were detected. Finally, we evaluated the DEGs shared between the 28ºC and 30ºC heat treatments and in the majority of cases decreased expression was observed with downregulated genes encoding an amastin-like surface protein, an amastin surface glycoprotein (LbrM.24.1270 and LbrM.27.0650) and ABC transporters (LbrM.15.0820, LbrM.29.0630 and LbrM.29.1750).

## DISCUSSION

Temperature affected the growth curve of *L. braziliensis*, with 30ºC heat treatment having the highest negative effect on parasite concentration (Fig. 1). This may be explained by the general response of promastigotes to higher temperatures as a consequence of host changes, as it has been reported that *in vitro* promastigotes lose their motility, become rounded and express HSPs in response to higher temperatures.^6^ ^,^ ^7^ Herein, we illustrated, the response of the growth curve at lower temperatures and found continuing growth of the parasite. However, the BLP was delayed at 24ºC compared with that at 28ºC and 30ºC. This perhaps reflect the effect of low temperatures on physiological changes in microorganisms and cells such as decreased membrane fluidity, decreased efficiency of transport proteins and a slower rate of growth.^16^

When evaluating changes at the chromosomal level, no variations in ploidy were detected in any of the 35 chromosomes either by analysing allele frequency; moreover, we found no significant variations when evaluating ploidy based on RNA-seq (Fig. 2). In other studies, it has been suggested that ploidy changes are a regular response to environmental change and drug resistance;^9^ ^,^ ^10^ ^,^ ^11^ ^,^ ^13^ ^,^ ^17^ however, we did not detect any ploidy changes indicating that variations in chromosome copy number may require a longer period of time to be fixed. Future studies should consider whether long-term temperature shifts impact on DNA ploidy. Some chromosomes, particularly chromosome 31, possessed a high number of genes with CNVs following a temperature shift compared with the control (Fig. 3). This chromosome is known to be supernumerary in many *Leishmania* species such as *L. major* and *L. peruviana*,^12^ and has a copy number of more than two in all sequenced species of *Leishmania*.^17^ Hence, this chromosome might be important for *Leishmania* adaptation and further studies are needed to investigate this.

We evaluated the genes with CNVs related with the enriched GO terms associated with every treatment. In first place, Elongation factor 1-alpha genes changes their copy number in the three temperature treatments, it is an important factor in protein transduction because it catalyses the GTP dependent binding of aminoacyl-tRNA to the A-site of ribosomes.^18^ We also found for the three treatments CNVs in genes related with cytoskeleton which are fundamental in cell shape, intracellular transport and morphological changes in *Leishmania*.^19^ For CNVs changes unique at each temperature, at 24 there was a high read depth in the copy number of genes that encode HSP70 (HSP70 is a chaperone protein that is expressed for environmental adaptation), and a putative transporter-like protein,^6^ ^,^ ^7^ but at the same time there was an increase in genes coding for ion transport and other membrane transport proteins that has been related with protozoan adaptation.^20^ Results from 28ºC and 30ºC treatments showed CNVs for genes that code ribosome subunits and signalling membrane proteins as well as heat shock proteins which are all important on *Leishmania* adaptation.^6^ ^,^ ^7^

When the CNVs were evaluated, along with their effects on gene expression, no correlations were found. Therefore, the variations in expression may be due to post-transcriptional regulatory mechanisms such as trans-splicing reactions of mRNAs and 3ʹ-polyadenylation,^21^ mRNA stability, translational control, and/or mRNA degradation.^22^ This conclusion is supported by the fact that at 30ºC, the chromosome with the highest proportion of DEGs was chromosome 12, which was the only chromosome that did not show CNVs in its genes (Fig. 3). However, we cannot assume that gene CNVs might be relevant to short-term adaptations to temperature for the slightly variation and the few days of the treatment, this variation could be stochastic so more studies should be done to infer the role that play CNVs in a temperature adaptation.

In addition, we observed a considerable number of DEGs as a possible response to the temperature treatments. One example was the overexpression of HSPs, which were upregulated principally at 30ºC but to a lesser extent at 24ºC. This may indicate that at more extreme temperatures, the impact of heat stress on the synthesis of HSPs is more noticeable. These upregulated HSPs play important roles in several cellular processes, such as protein folding, assembly, trafficking, activity and degradation.^23^ Specifically, the HSPs identified in this study are contained within a complex, since the activator HSP90 ATPase is fundamental for the activation of HSP90,^23^ which forms the centre of a chaperone complex known as the HSP90 foldosome. Together with other co-chaperones, such as HSP70, HSP40 and stress-inducible protein 1 (Sti1), HSP90 is implicated in certain functions such as transduction signalling and cell cycle control.^24^ In addition, each HSP has other functions, such as, RNA stability and translational control and, in the case of HSP70, in transcript stability.^24^ Moreover, these proteins also play other important roles that directly affect the cell cycle, since some of the HSPs, such as HSP90 and several associated chaperones and co-chaperones, are involved in natural modulation pathways that include protein kinases. Examples of this include being substrates for MAP kinase 1, which is crucial for the intracellular survival of *Leishmania*; casein kinase 1.2 catalysing HSP90 phosphorylation to promote promastigote growth;^25^ and the interaction between HSP90 and Sti1 to promote the fast-growing insect and mammalian host stages of the parasite.^26^

Other genes found to be expressed in association with the three treatments were those encoding the amastin surface-like proteins, which showed a pattern of downregulation under the three temperatures compared with the control. These proteins are members of a multigene family encoding glycoproteins that are important components of the parasite surface, being involved in host-parasite interactions and playing a fundamental role during infection.^27^ Therefore, a reduction in the expression of these genes could affect these processes, as was reported by Cardoso de Paiva et al.,^27^ who described that amastin knockdown generated a reduction in the viability of intracellular amastigotes.^27^

As reported in the current study, other genes that were underexpressed at all of the test temperatures were those that encode the ABC transporters,^28^ which have been reported to be important in the infection process.^28^ ^,^ ^29^ The transcriptome profile described above shared similarities with that reported by Rastrojo et al.,^30^ in which *L. major* promastigotes were exposed to heat shock at 37ºC for two hours. They reported upregulation of genes that encode for several HSPs, which was consistent with our results; in addition, the most downregulated transcript in their study was an ABC transporter, which was also detected in our study, and the amastin-encoding genes were also found to be downregulated in both studies.^30^ These results confirmed that these genes are sensitive to temperature shifts.

In conclusion, our study provides evidence that *L. braziliensis* promastigotes exhibit characteristic changes in DNA and mRNA response to short-term temperature stress *in vitro*. This evidence included changes in the growth curves, CNVs in 3% of the genes evaluated at each temperature, up- or downregulation of a range of genes associated with temperature stress (HSPs were upregulated at 30ºC, and amastin-like proteins and ABC transporters were downregulated at the three temperatures) and variations in expression of many genes associated with the GO terms of cellular, biosynthetic and biological processes. These findings revealed the great impact of temperature on the transcriptome profile, leading to rapid and efficient cellular responses. Furthermore, we found that temperature has a negative effect on the growth curves of *L. braziliensis*, at least in the short-term. Our findings provide the first insights into the genomic and transcriptomic changes in *L. braziliensis* following temperature shifts, confirming the important role played by this abiotic factor in biological processes in the parasite over the short-term. One limitation of this study was the use of promastigotes under controlled conditions. Future studies should consider the impact of temperature shifts on promastigotes infecting sandflies and on amastigotes in the mammalian host to determine the holistic factors impacting the dynamics of the genome and transcriptome of *Leishmania* parasites.

The data set generated during the current study was deposited at DDBJ/ENA/GenBank under the accession number PRJEB31852 (ERP114463).
