# Supplementary material for: Minor temperature shifts do not affect chromosomal ploidy but cause transcriptomic changes in Leishmania braziliensis promastigotes in vitro
Source: Mem Inst Oswaldo Cruz. 2020 Apr 27;115:e190413. doi: 10.1590/0074-02760190413 (PMC7184772; doi:10.1590/0074-02760190413)
Supplement: Supplementary file 1 [file 1678-8060-mioc-115-e190413-s1.pdf]

TABLE I

DNA-seq statistics

DNA Sample	Depth	Total reads	Mapped and paired reads	Aligned reads (%)
Lb_24	74.6X	18392456	18067438	98.233
Lb_26	91.1X	25000050	23206035	92.820
Lb_28	77.1X	19398414	18816976	97.003
Lb_30	87.1X	22591350	21446012	94.930

TABLE II

Genes resulted by the DNA sequence of *Leishmania braziliensis* under 24ºC with a significative increase or decrease in copy number variation (CNV) compared with the control temperature of 26ºC

Chromosome	Total genes	Genes with CNVs	Gene IDs with increase CNVs	Gene IDs with decrease CNVs
1	78	5	LbrM.01.0170 LbrM.01.0270 LbrM.01.0640 LbrM.01.0740 LbrM.01.0760	
2	68	5	LbrM.02.0070 LbrM.02.0090 LbrM.02.0140 LbrM.02.0220 LbrM.02.0290	
3	96	0		
4	125	7	LbrM.04.0210 LbrM.04.0220 LbrM.04.0230 LbrM.04.0650 LbrM.04.1080	LbrM.04.0740 LbrM.04.1020
5	129	7	LbrM.05.0380 LbrM.05.0390 LbrM.05.snoRNA3	LbrM.05.0520 LbrM.05.snRNA1 LbrM.05.snoRNA1 LbrM.05.snoRNA2
6	130	8	LbrM.06.0090 LbrM.06.0100 LbrM.06.0330 LbrM.06.0580 LbrM.06.0590 LbrM.06.1050 LbrM.06.1170 LbrM.06.1240	
7	129	3	LbrM.07.0510 LbrM.07.1060 LbrM.07.1080	
8	97	0		
9	169	6	LbrM.09.0180 LbrM.09.1070 LbrM.09.1220 LbrM.09.1490	LbrM.09.0170 LbrM.09.tRNA3
10	156	6	LbrM.10.0490 LbrM.10.1260	LbrM.10.1070 LbrM.10.1071 LbrM.10.1080 LbrM.10.1090
11	123	2	LbrM.11.0760	LbrM.11.0220
12	94	1	LbrM.12.0710	
13	158	4	LbrM.13.0190 LbrM.13.0200 LbrM.13.1330	LbrM.13.1070
14	169	10	LbrM.14.0060 LbrM.14.0070 LbrM.14.0150 LbrM.14.0210 LbrM.14.0660 LbrM.14.1110 LbrM.14.1120 LbrM.14.1300 LbrM.14.1310 LbrM.14.1510	
15	164	3	LbrM.15.1080 LbrM.15.1280	LbrM.15.tRNA2
16	174	4	LbrM.16.0030 LbrM.16.1510	LbrM.16.0470 LbrM.16.0790
17	166	5	LbrM.17.0090 LbrM.17.0110 LbrM.17.1440 LbrM.17.1570	LbrM.17.tRNA2
18	171	8	LbrM.18.0850 LbrM.18.1270 LbrM.18.1550 LbrM.18.1620	LbrM.18.0450 LbrM.18.0460 LbrM.18.0470 LbrM.18.1720
19	192	4	LbrM.19.0100 LbrM.19.1180 LbrM.19.1730	LbrM.19.0950
20.1	451	15	LbrM.20.0620 LbrM.20.0780 LbrM.20.2480 LbrM.20.4280 LbrM.20.4290	LbrM.20.0720 LbrM.20.0760 LbrM.20.1.snoRNA2 LbrM.20.1.tRNA3 LbrM.20.1.tRNA4 LbrM.20.1.tRNA5 LbrM.20.1010 LbrM.20.1110 LbrM.20.1460 LbrM.20.4300
20.2	181	3	LbrM.20.5550 LbrM.20.5910	LbrM.20.5530
21	212	3	LbrM.21.0620 LbrM.21.tRNA1	LbrM.21.1140
22	163	6	LbrM.22.0520 LbrM.22.1330 LbrM.22.1370 LbrM.22.1560	LbrM.22.0330 LbrM.22.0640
23	217	0		
24	248	1	LbrM.24.1600	
25	203	9	LbrM.25.0320 LbrM.25.0340 LbrM.25.0410 LbrM.25.1010 LbrM.25.1290 LbrM.25.1690 LbrM.25.1730 LbrM.25.1960	LbrM.25.0990
26	278	7	LbrM.26.0680 LbrM.26.1890 LbrM.26.snoRNA13 LbrM.26.snoRNA6	LbrM.26.snoRNA10 LbrM.26.snoRNA4 LbrM.26.snoRNA9

CC BY

Chromosome	Total genes	Genes with CNVs	Gene IDs with increase CNVs	Gene IDs with decrease CNVs
27	286	13	LbrM.27.0250 LbrM.27.0810 LbrM.27.2330 LbrM.27.2751 LbrM.27.2810 LbrM.27.2830	LbrM.27.0760 LbrM.27.1480 LbrM.27.1730 LbrM.27.1920 LbrM.27.1940 LbrM.27.1950 LbrM.27.2530
28	328	9	LbrM.28.1020 LbrM.28.2100 LbrM.28.2990 LbrM.28.3240	LbrM.28.0220 LbrM.28.0850 LbrM.28.1080 LbrM.28.1660 LbrM.28.2970
29	291	3	LbrM.29.2700 LbrM.29.2720	LbrM.29.1690
30	383	8	LbrM.30.0440 LbrM.30.1491 LbrM.30.3640 LbrM.30.3760	LbrM.30.0770 LbrM.30.1790 LbrM.30.2740 LbrM.30.2970
31	376	19	LbrM.31.0280 LbrM.31.0380 LbrM.31.0920 LbrM.31.1330 LbrM.31.1660 LbrM.31.1670 LbrM.31.1800 LbrM.31.1840 LbrM.31.2190 LbrM.31.2920 LbrM.31.3290 LbrM.31.3480 LbrM.31.3490	LbrM.31.0080 LbrM.31.2250 LbrM.31.2770 LbrM.31.ncRNA1 LbrM.31.snRNA1 LbrM.31.tRNA1
32	428	5	LbrM.32.2500 LbrM.32.4200	LbrM.32.0630 LbrM.32.0780 LbrM.32.3420
33	367	14	LbrM.33.0960 LbrM.33.1580 LbrM.33.2100 LbrM.33.tRNA1 LbrM.33.tRNA2	LbrM.33.0330 LbrM.33.0920 LbrM.33.0950 LbrM.33.0990 LbrM.33.1010 LbrM.33.1570 LbrM.33.1690 LbrM.33.3080 LbrM.33.ncRNA3
34	545	8	LbrM.34.0020 LbrM.34.0050 LbrM.34.0530 LbrM.34.0540	LbrM.34.1100 LbrM.34.1110 LbrM.34.3460 LbrM.34.4460
35	761	15	LbrM.35.0270 LbrM.35.3760 LbrM.35.7310 LbrM.35.snoRNA6	LbrM.35.1320 LbrM.35.1790 LbrM.35.1960 LbrM.35.4000 LbrM.35.4800 LbrM.35.6690 LbrM.35.snRNA1 LbrM.35.snoRNA1 LbrM.35.snoRNA3 LbrM.35.snoRNA4 LbrM.35.snoRNA5

TABLE III

Genes resulted by the DNA sequence of *Leishmania braziliensis* under 28ºC with a significative increase or decrease in copy number variation (CNV) compared with the control temperature of 26ºC

Chromosome	Total genes	Genes with CNVs	Gene IDs with increase CNVs	Gene IDs with decrease CNVs
1	78	6	LbrM.01.0170 LbrM.01.0300 LbrM.01.0640 LbrM.01.0680 LbrM.01.0740 LbrM.01.0760	
2	68	8	LbrM.02.0140 LbrM.02.0160 LbrM.02.0220 LbrM.02.0290 LbrM.02.0650 LbrM.02.0660 LbrM.02.0680	LbrM.02.0420
3	96	1	LbrM.03.ncRNA3	
4	125	5	LbrM.04.0210 LbrM.04.0220 LbrM.04.0230	LbrM.04.0950 LbrM.04.1020
5	129	4	LbrM.05.0380 LbrM.05.0390 LbrM.05.0990	LbrM.05.snoRNA1
6	130	10	LbrM.06.0100 LbrM.06.0110 LbrM.06.0200 LbrM.06.0330 LbrM.06.0590 LbrM.06.0680 LbrM.06.0940 LbrM.06.1170 LbrM.06.ncRNA1	LbrM.06.1280
7	129	3	LbrM.07.0150 LbrM.07.0155 LbrM.07.1060	
8	97	4	LbrM.08.0030 LbrM.08.0500 LbrM.08.0630 LbrM.08.0670	
9	169	5	LbrM.09.0770 LbrM.09.1070	LbrM.09.0070 LbrM.09.0170 LbrM.09.tRNA3
10	156	6	LbrM.10.0250 LbrM.10.0620	LbrM.10.0970 LbrM.10.1070 LbrM.10.1080 LbrM.10.1090
11	123	1		LbrM.11.0910
12	94	1	LbrM.12.0710	
13	158	2	LbrM.13.0190 LbrM.13.0200	
14	169	11	LbrM.14.0010 LbrM.14.0030 LbrM.14.0060 LbrM.14.0200 LbrM.14.0220 LbrM.14.0250 LbrM.14.0660 LbrM.14.1110 LbrM.14.1120 LbrM.14.1300 LbrM.14.1310	
15	164	4	LbrM.15.0540 LbrM.15.1080 LbrM.15.1280 LbrM.15.1421	
16	174	2	LbrM.16.1510 LbrM.16.1520	
17	166	4	LbrM.17.0090 LbrM.17.0110 LbrM.17.1440 LbrM.17.1530	
18	171	5		LbrM.18.0010 LbrM.18.0450 LbrM.18.0460 LbrM.18.0470 LbrM.18.1720
19	192	7	LbrM.19.0120 LbrM.19.1180 LbrM.19.1730	LbrM.19.0340 LbrM.19.0350 LbrM.19.0360 LbrM.19.0950
20.1	451	17	LbrM.20.0620 LbrM.20.0780 LbrM.20.4290 LbrM.20.4340	LbrM.20.1.snoRNA2 LbrM.20.1.tRNA3 LbrM.20.1.tRNA4 LbrM.20.1.tRNA8 LbrM.20.1090 LbrM.20.1100 LbrM.20.1460 LbrM.20.2870 LbrM.20.2960 LbrM.20.3230 LbrM.20.3900 LbrM.20.3950 LbrM.20.4000
20.2	181	2	LbrM.20.5550	LbrM.20.5590
21	212	5	LbrM.21.0180 LbrM.21.0620 LbrM.21.2020 LbrM.21.tRNA1	LbrM.21.1820
22	163	5	LbrM.22.0520 LbrM.22.1330 LbrM.22.1380 LbrM.22. ncRNA2	LbrM.22.0040
23	217	8	LbrM.23.1120 LbrM.23.1620	LbrM.23.0330 LbrM.23.0670 LbrM.23.1610 LbrM.23. tRNA10 LbrM.23.tRNA6 LbrM.23.tRNA8
24	248	2	LbrM.24.1990	LbrM.24.1590
25	203	7	LbrM.25.0340 LbrM.25.0410 LbrM.25.0900 LbrM.25.1010 LbrM.25.1290	LbrM.25.0590 LbrM.25.0610
26	278	6	LbrM.26.2060 LbrM.26.snoRNA13 LbrM.26.snoRNA14 LbrM.26.snoRNA6	LbrM.26.0890 LbrM.26.2610
27	286	12	LbrM.27.0260 LbrM.27.2330 LbrM.27.2751 LbrM.27.2810 LbrM.27.2830	LbrM.27.0070 LbrM.27.0760 LbrM.27.0830 LbrM.27.1040 LbrM.27.1730 LbrM.27.1950 LbrM.27.2260
28	328	6	LbrM.28.2090 LbrM.28.2100 LbrM.28.2760 LbrM.28.3240	LbrM.28.0220 LbrM.28.2980
29	291	4	LbrM.29.1010 LbrM.29.2700 LbrM.29.2720	LbrM.29.2430
30	383	9	LbrM.30.0440 LbrM.30.1491 LbrM.30.1720 LbrM.30.3640 LbrM.30.3760	LbrM.30.0450 LbrM.30.1790 LbrM.30.2420 LbrM.30.2970
31	376	14	LbrM.31.0570 LbrM.31.0920 LbrM.31.1660 LbrM.31.1670 LbrM.31.1800 LbrM.31.1840 LbrM.31.2220	LbrM.31.0010 LbrM.31.0080 LbrM.31.1210 LbrM.31.1260 LbrM.31.2940 LbrM.31.3270 LbrM.31.tRNA4
32	428	4	LbrM.32.2500 LbrM.32.3860	LbrM.32.1660 LbrM.32.1960
33	367	8	LbrM.33.1880 LbrM.33.2100	LbrM.33.0020 LbrM.33.0920 LbrM.33.0950 LbrM.33.0990 LbrM.33.1570 LbrM.33.ncRNA3
34	545	9	LbrM.34.0050 LbrM.34.0530 LbrM.34.0540	LbrM.34.1110 LbrM.34.1790 LbrM.34.2130 LbrM.34.3430 LbrM.34.3440 LbrM.34.3470
35	761	11	LbrM.35.3760 LbrM.35.7170 LbrM.35.snoRNA2 LbrM.35. snoRNA5 LbrM.35.snoRNA6	LbrM.35.0660 LbrM.35.1010 LbrM.35.1790 LbrM.35.1960 LbrM.35.snRNA1 LbrM.35.snoRNA4

TABLE IV

Genes resulted by the DNA sequence of *Leishmania braziliensis* under 30ºC with a significative increase or decrease in copy number variation (CNV) compared with the control temperature of 26ºC

Chromosome	Total genes	Genes with CNVs	Gene IDs with increase CNVs	Gene IDs with decrease CNVs
1	78	4	LbrM.01.0170 LbrM.01.0230 LbrM.01.0270 LbrM.01.0640	
2	68	7	LbrM.02.0140 LbrM.02.0160 LbrM.02.0220 LbrM.02.0290	LbrM.02.0300 LbrM.02.0420 LbrM.02.0550
3	96	4	LbrM.03.0360 LbrM.03.0660 LbrM.03.0930 LbrM.03.ncRNA3	
4	125	6	LbrM.04.0210 LbrM.04.0220 LbrM.04.0230	LbrM.04.0200 LbrM.04.0750 LbrM.04.0950
5	129	5	LbrM.05.0380 LbrM.05.0390 LbrM.05.snRNA1 LbrM.05.snoRNA3	LbrM.05.snoRNA1
6	130	3	LbrM.06.0110	LbrM.06.0040 LbrM.06.0720
7	129	4	LbrM.07.0155 LbrM.07.1060 LbrM.07.1080	LbrM.07.0550
8	97	2	LbrM.08.0050 LbrM.08.0670	
9	169	9	LbrM.09.0010 LbrM.09.0180 LbrM.09.0190 LbrM.09.1070 LbrM.09.1220	LbrM.09.0070 LbrM.09.0170 LbrM.09.0650 LbrM.09.tRNA3
10	156	2	LbrM.10.0530	LbrM.10.1080
11	123	2		LbrM.11.tRNA1 LbrM.11.tRNA2
12	94	0		
13	158	3	LbrM.13.0190 LbrM.13.0740	LbrM.13.0200
14	169	5	LbrM.14.1110 LbrM.14.1300 LbrM.14.1310	LbrM.14.0390 LbrM.14.1190
15	164	4	LbrM.15.0450 LbrM.15.0730 LbrM.15.1080 LbrM.15.1280	
16	174	5	LbrM.16.1370 LbrM.16.1510 LbrM.16.1520	LbrM.16.0470 LbrM.16.0790
17	166	3	LbrM.17.0090 LbrM.17.0980	LbrM.17.1620
18	171	6		LbrM.18.0450 LbrM.18.0460 LbrM.18.0470 LbrM.18.0490 LbrM.18.1150 LbrM.18.1720
19	192	4	LbrM.19.1170 LbrM.19.1180 LbrM.19.1730	LbrM.19.0950
20.1	451	17	LbrM.20.0620 LbrM.20.0780 LbrM.20.1050 LbrM.20.1070 LbrM.20.1080 LbrM.20.1630 LbrM.20.2370 LbrM.20.2410 LbrM.20.4290	LbrM.20.0650 LbrM.20.0930 LbrM.20.0950 LbrM.20.1.snoRNA2 LbrM.20.1.tRNA10 LbrM.20.1.tRNA4 LbrM.20.1.tRNA8 LbrM.20.1.tRNA9
20.2	181	3	LbrM.20.5550	LbrM.20.5530 LbrM.20.5590
21	212	3	LbrM.21.0440 LbrM.21.0620	LbrM.21.0610
22	163	5	LbrM.22.0520 LbrM.22.1270 LbrM.22.1410 LbrM.22.1550 LbrM.22.ncRNA2	
23	217	11		LbrM.23.0621 LbrM.23.0670 LbrM.23.1000 LbrM.23.1110 LbrM.23.1130 LbrM.23.1830 LbrM.23.rRNA1 LbrM.23.tRNA10 LbrM.23.tRNA3 LbrM.23.tRNA6 LbrM.23.tRNA8
24	248	3	LbrM.24.0200 LbrM.24.1990	LbrM.24.1590
25	203	5	LbrM.25.0320 LbrM.25.0340 LbrM.25.1010 LbrM.25.1690	LbrM.25.0610
26	278	9	LbrM.26.0020 LbrM.26.0180 LbrM.26.1070 LbrM.26.snoRNA11 LbrM.26.snoRNA13 LbrM.26.snoRNA14 LbrM.26.snoRNA6 LbrM.26.snoRNA7	LbrM.26.snoRNA2
27	286	12	LbrM.27.0140 LbrM.27.0460 LbrM.27.0850 LbrM.27.2330 LbrM.27.2751 LbrM.27.2810 LbrM.27.2830	LbrM.27.0760 LbrM.27.1730 LbrM.27.1920 LbrM.27.2360 LbrM.27.2450
28	328	7	LbrM.28.0650 LbrM.28.2100 LbrM.28.2760 LbrM.28.3240	LbrM.28.1030 LbrM.28.1080 LbrM.28.2970
29	291	6	LbrM.29.2860	LbrM.29.0750 LbrM.29.1370 LbrM.29.1690 LbrM.29.2340 LbrM.29.2900
30	383	10	LbrM.30.0440 LbrM.30.1491 LbrM.30.2620 LbrM.30.3230 LbrM.30.3640 LbrM.30.3760 LbrM.30.tRNA1	LbrM.30.0450 LbrM.30.1790 LbrM.30.2970
31	376	27	LbrM.31.0280 LbrM.31.0290 LbrM.31.0380 LbrM.31.0430 LbrM.31.0530 LbrM.31.0920 LbrM.31.1430 LbrM.31.1670 LbrM.31.1840 LbrM.31.2190 LbrM.31.2260 LbrM.31.2740 LbrM.31.3290 LbrM.31.3330 LbrM.31.3370 LbrM.31.3420 LbrM.31.3620 LbrM.31.3630	LbrM.31.0080 LbrM.31.1250 LbrM.31.1380 LbrM.31.1520 LbrM.31.2001 LbrM.31.2120 LbrM.31.2250 LbrM.31.3640 LbrM.31.tRNA4
32	428	3	LbrM.32.2200 LbrM.32.2500 LbrM.32.4000	
33	367	9	LbrM.33.0340 LbrM.33.0930 LbrM.33.0960	LbrM.33.0330 LbrM.33.0920 LbrM.33.0950 LbrM.33.0990 LbrM.33.1010 LbrM.33.ncRNA3
34	545	11	LbrM.34.0170 LbrM.34.0530 LbrM.34.0540 LbrM.34.1670 LbrM.34.2470 LbrM.34.snoRNA1	LbrM.34.1100 LbrM.34.1110 LbrM.34.1790 LbrM.34.2160 LbrM.34.3350
35	761	19	LbrM.35.0120 LbrM.35.3760 LbrM.35.5260 LbrM.35.6650 LbrM.35.7170 LbrM.35.snoRNA2 LbrM.35.snoRNA6	LbrM.35.1140 LbrM.35.1570 LbrM.35.1960 LbrM.35.2730 LbrM.35.2800 LbrM.35.3820 LbrM.35.5990 LbrM.35.snRNA1 LbrM.35.snoRNA4 LbrM.35.snoRNA5 LbrM.35.tRNA1 LbrM.35.tRNA6

TABLE V

RNA-seq statistics

RNA sample*	Depth**	Total reads	Mapped and paired reads	Aligned reads (%)
Lb_1_24	67.3X [63.3-71.0]	19394788	18894068	97.418
Lb_2_24		18663462	18169842	97.355
Lb_3_24		17365542	16858012	97.077
Lb_4_24		19740722	19159978	97.058
Lb_1_26	156.4X [143.7-174.7]	43731670	39524741	90.380
Lb_2_26		42357932	38371252	90.590
Lb_3_26		47035840	42626338	90.630
Lb_4_26		52807822	47748465	90.420
Lb_1_28	71.9X [68.3-74.7]	19690762	19189066	97.452
Lb_2_28		18378510	17969192	97.773
Lb_3_28		6318384	6163752	97.553
Lb_4_28		20218216	19720138	97.536
Lb_1_30	66.7X [60.4-76.6]	21155634	20663116	97.672
Lb_2_30		15955118	15545860	97.435
Lb_3_30		16223390	15766176	97.182
Lb_4_30		16380050	15961560	97.445

*: the four replicates assessed (two biological and two technical) per treatment indicated between the letters "Lb" (abbreviation for *Leishmania braziliensis*) and the last number (the temperature assessed); for instance, Lb_1_24 is the first replicate at the temperature of 24ºC. **: these values correspond to the average depth between the replicates per temperature and the values inside the parenthesis are the depth's range of the replicates.

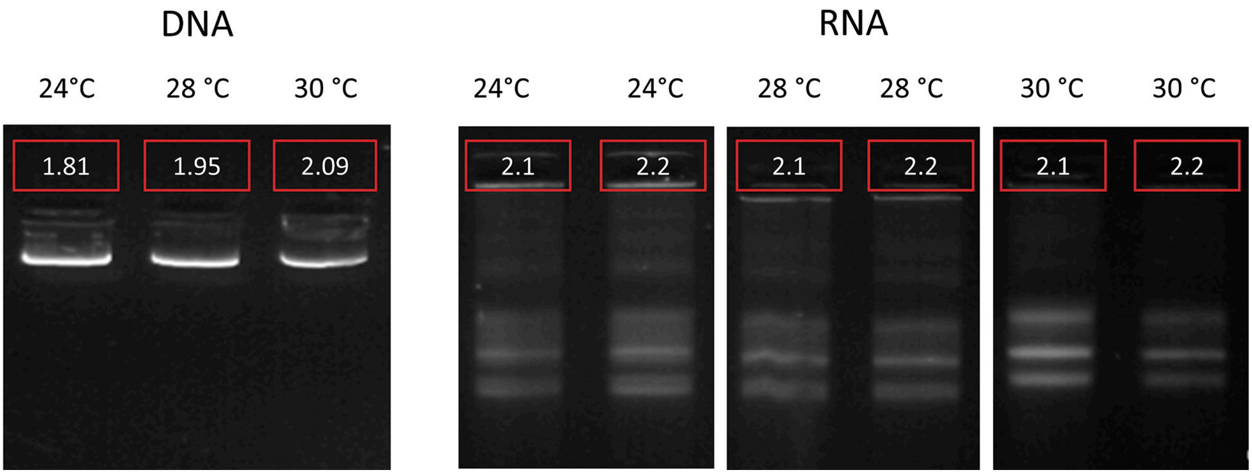

Fig. 1: the integrity of the DNA and RNA samples for all of the treatments, as assessed by electrophoresis on a 1% agarose gel. For the RNA, both biological replicates per treatment are labelled with the temperature number. The A_{260}/A_{280} ratio for each sample is shown inside the red rectangles.

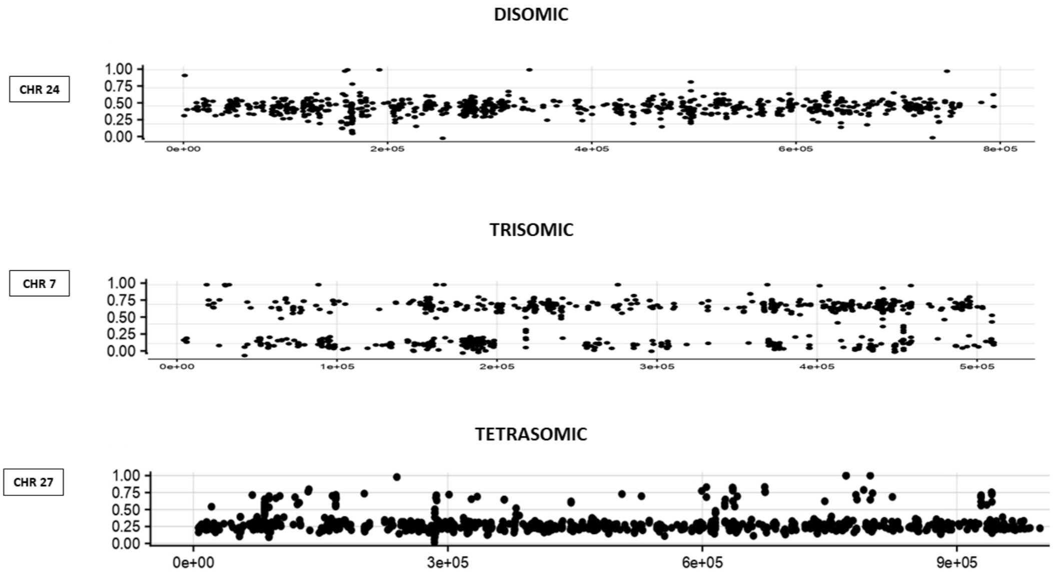

Fig. 2: allele distribution frequency of specific chromosomes by ploidy difference. Chromosome 24 is disomic, chromosome 7 is trisomic and chromosome 27 is tetrasomic.

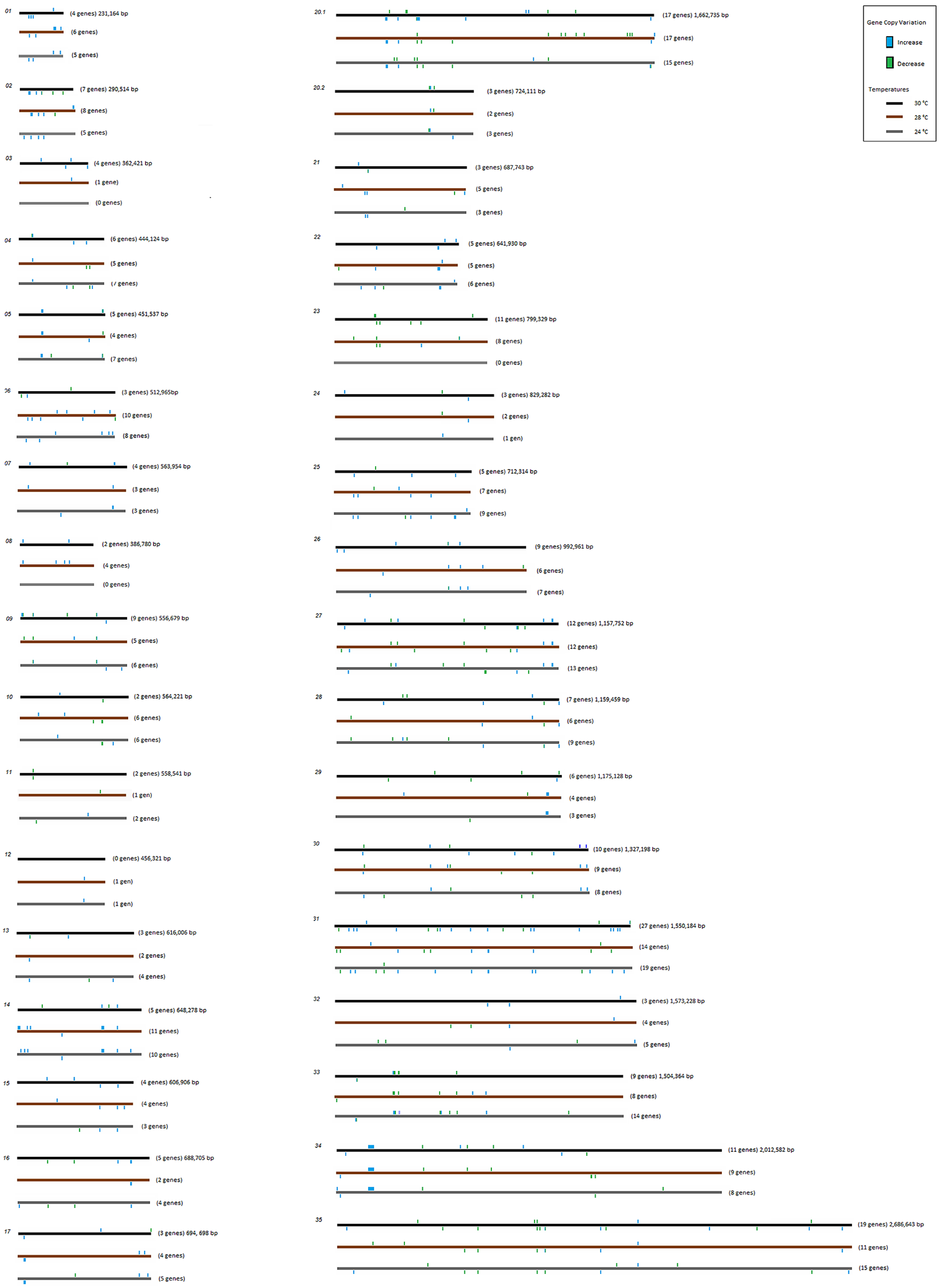

Fig. 3: genomic distribution of multicopy genes (genes with copy number variations) across the chromosomes of *Leishmania braziliensis* under different temperatures. The colour of the multicopy genes indicates an increase (blue) or decrease (green) compared with the control temperature of 26ºC. The genomic results at 30ºC, 28ºC and 24ºC are represented by the chromosomes and the colours black, brown and grey, respectively.

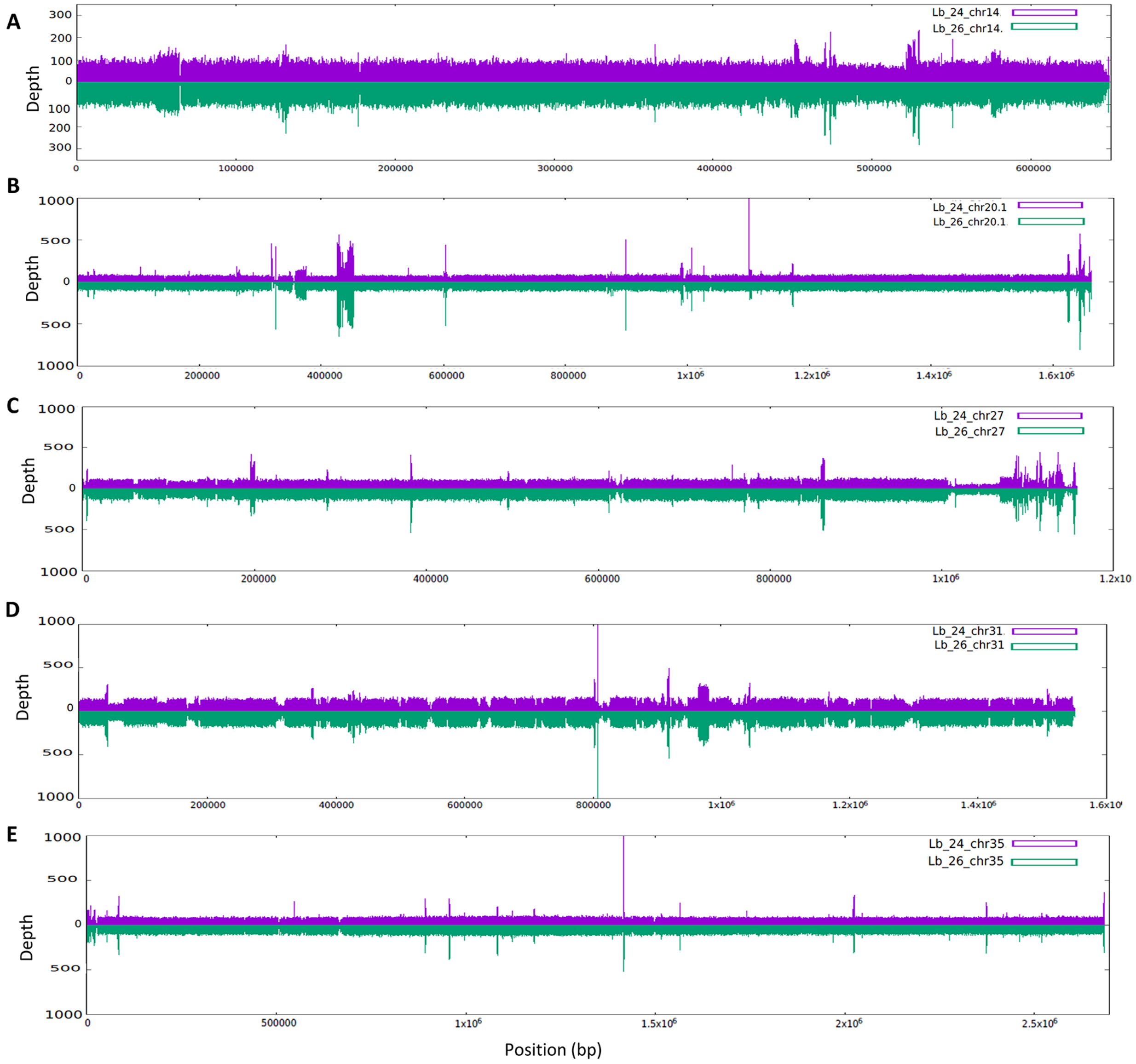

Fig. 4: read depth distribution of chromosomes with higher numbers of genes with copy number variations (CNVs) at 24ºC (purple bars) compared with 26ºC (green bars). The chromosomes represented are (A) 14, (B) 20.1, (C) 27, (D) 31 and (E) 35. The y axis is the normalised read depth sequencing and the x axis is the chromosome position in base pairs.

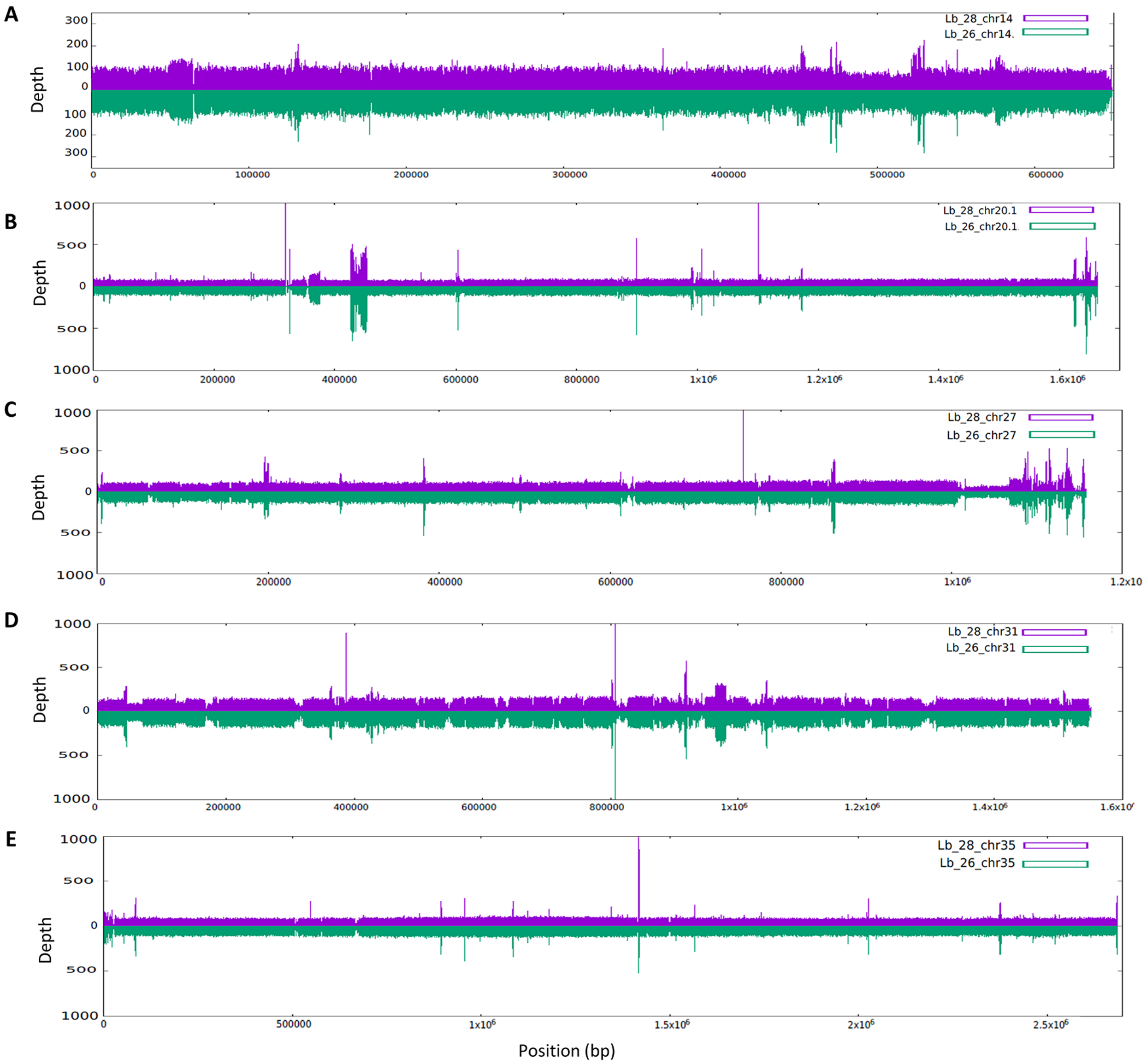

Fig. 5: read depth distribution of chromosomes with higher numbers of genes with copy number variations (CNVs) at 28ºC (purple bars) compared with 26ºC (green bars). The chromosomes represented are (A) 14, (B) 20.1, (C) 27, (D) 31 and (E) 35. The y axis is the normalised read depth sequencing and the x axis is the chromosome position in base pairs.

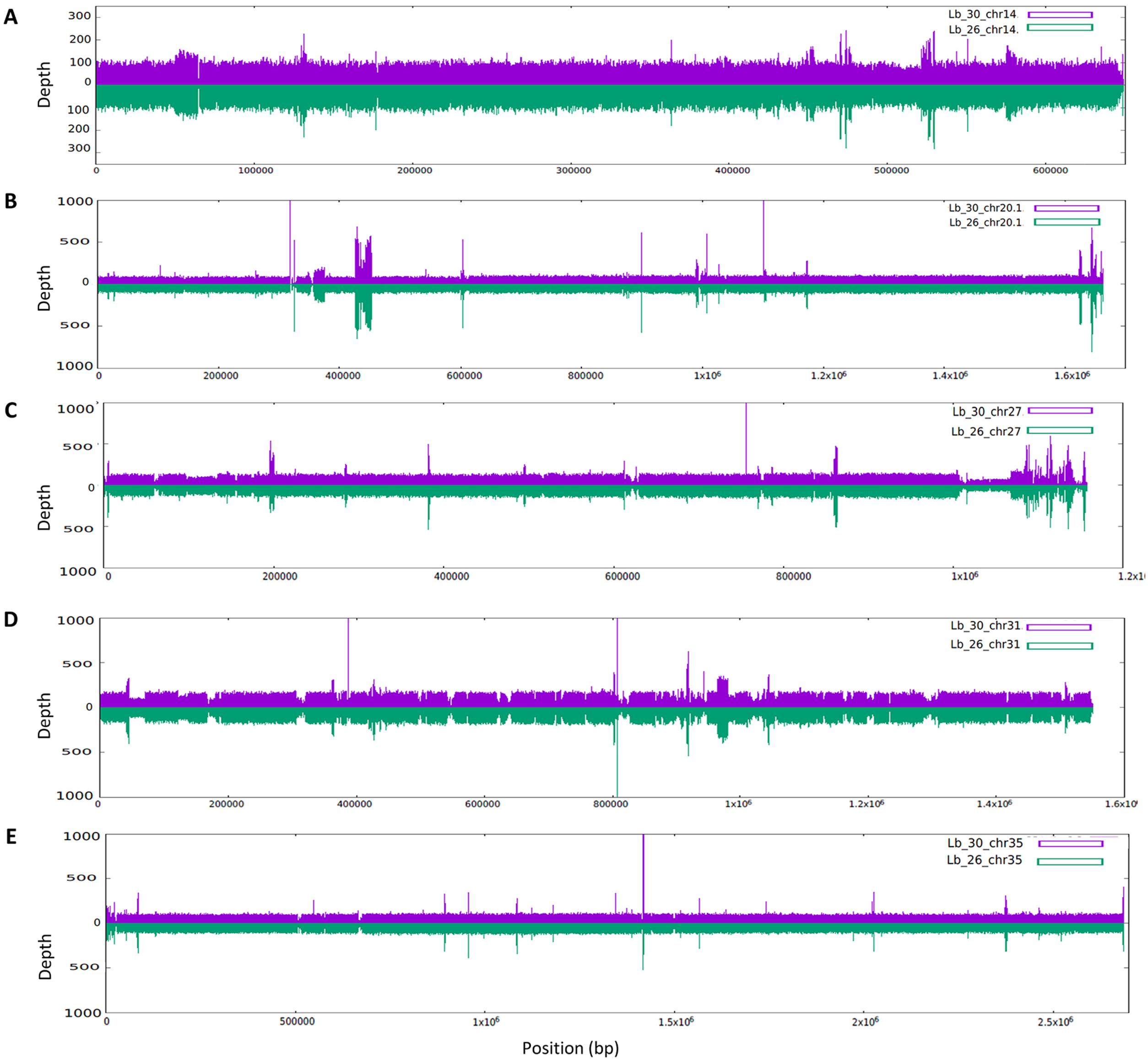

Fig. 6: read depth distribution of chromosomes with higher numbers of genes with copy number variations (CNVs) at 30ºC (purple bars) compared with 26ºC (green bars). The chromosomes represented are (A) 14, (B) 20.1, (C) 27, (D) 31 and (E) 35. The y axis is the normalized read depth sequencing and the x axis is the chromosome position in base pairs.

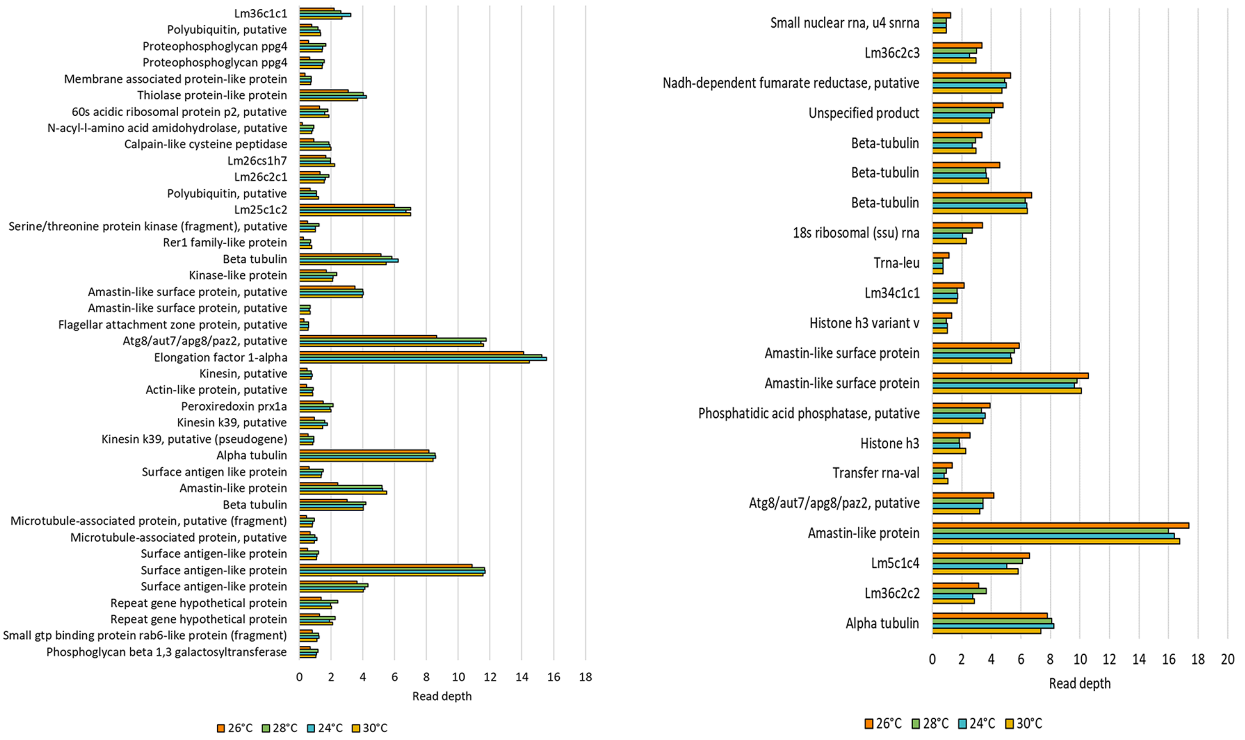

Fig. 7: read depth of genes shared by the three temperatures compared with the control temperature. The genes of increased read depth compared with the control are represented on the left and the genes of decreased read depth are shown on the right.

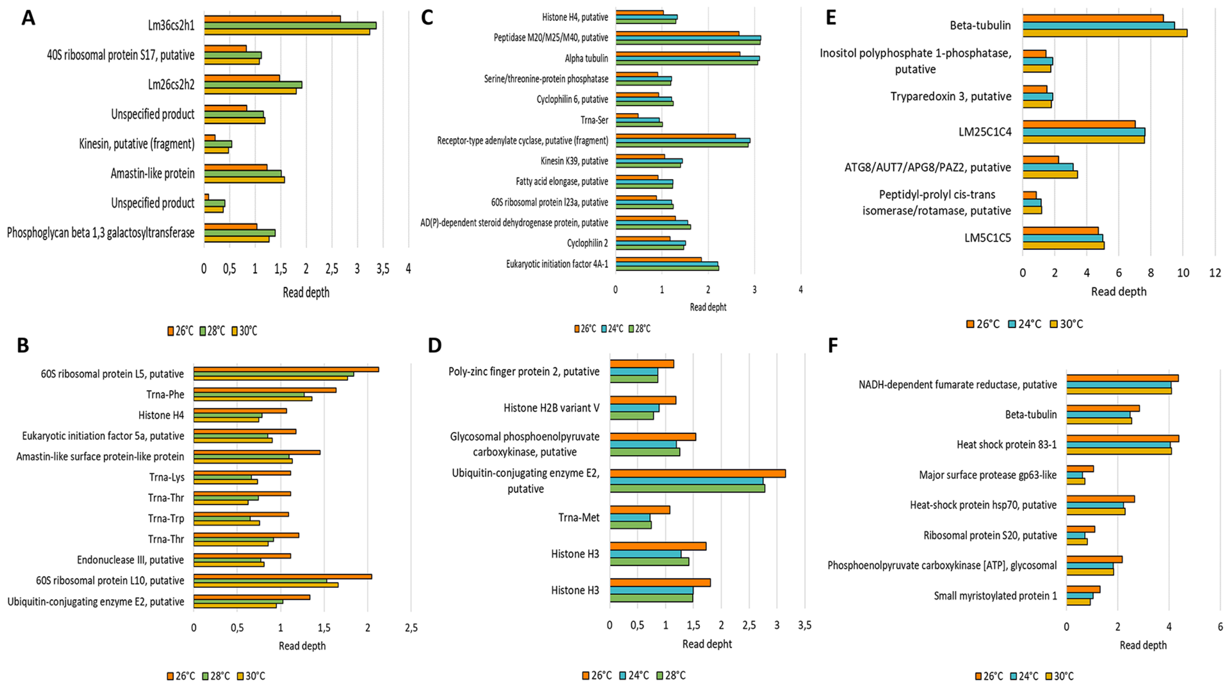

Fig. 8: read depth of genes shared by only two temperatures compared with the control temperature. On the left are the unique genes shared by 28ºC and 30ºC, in the middle are the genes shared by 24ºC and 28ºC and on the right are the genes shared by only 24ºC and 30ºC. The genes of increased read depth compared with the control are represented in the upper three graphs and the genes of decreased read depth are shown in the lower three graphs.

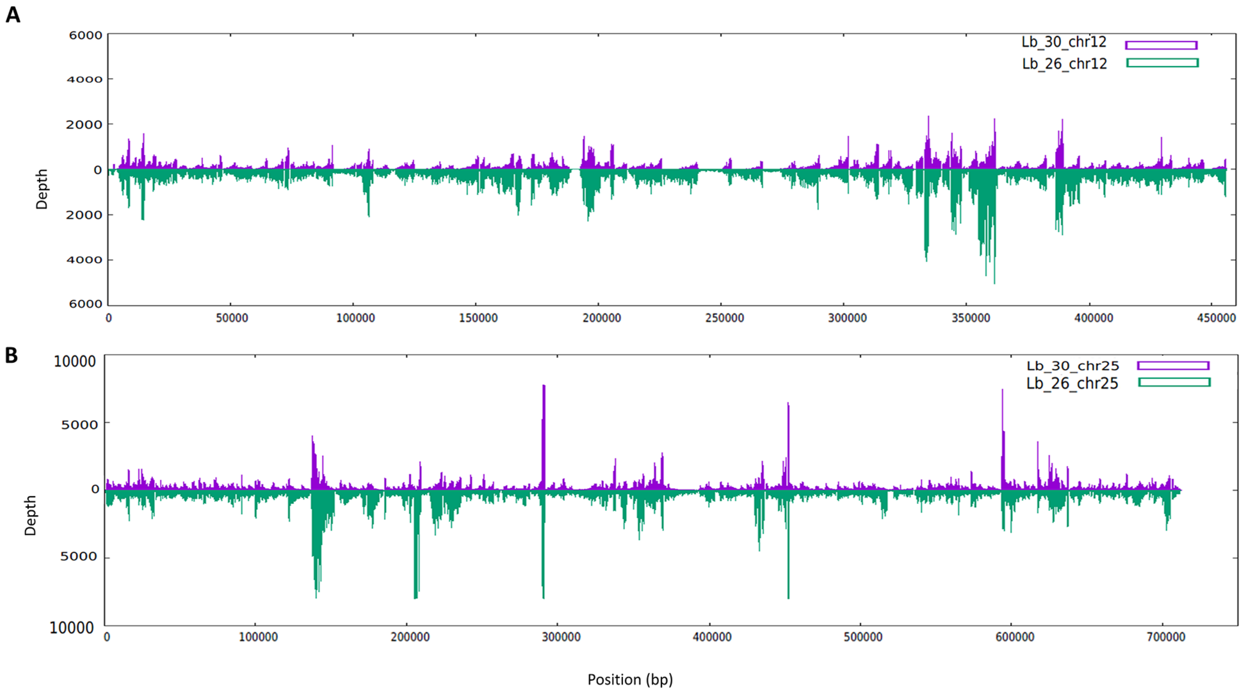

Fig. 9: read depth distribution of the chromosomes with the higher number of differentially expressed genes. (A) Representation of chromosome 12, which had the highest number of differentially expressed genes (DEGs) downregulated at 30ºC. Comparison of the read depth distribution over chromosome 12 between the results obtained at 30ºC (purple bars) and 26ºC (green bars). (B) Read depth distribution over chromosome 25, the chromosome with the highest number of DEGs upregulated at 30ºC. Comparison between the results obtained at 30°C (purple bars) and 26ºC (green bars). The y axis shows the normalised read depths from sequencing and the x axis shows the chromosome position in base pairs.
